# Immunological and metabolic characteristics of the Omicron variants infection

**DOI:** 10.1038/s41392-022-01265-8

**Published:** 2023-01-21

**Authors:** Jiejie Geng, Xu Yang, Kun Wang, Ke Wang, Ruo Chen, Zhi-Nan Chen, Chuan Qin, Guizhen Wu, Youchun Wang, Ke Xu, Peng Du, Jiangning Liu, Shirui Chen, Tao Zhang, Xiuxuan Sun, Ting Guo, Ying Shi, Zheng Zhang, Ding Wei, Peng Lin, Qingyi Wang, Jing Yuan, Jiuxin Qu, Jin Zou, Yingxia Liu, Hongzhou Lu, Ping Zhu, Huijie Bian, Liang Chen

**Affiliations:** 1 Department of Cell Biology of National Translational Science Center for Molecular Medicine and Department of Clinical Immunology of Xijing Hospital, Fourth Military Medical University, Xi’an, 710032 China; 2 Key Laboratory of Human Disease Comparative Medicine, Chinese Ministry of Health, Key Laboratory for Animal Models of Emerging and Remerging Infectious Diseases, Institute of Laboratory Animal Science, Chinese Academy of Medical Sciences and Peking Union Medical College, Beijing, 100871 China; 3 MHC Key Laboratory of Biosafety, National Institute for Viral Disease Control and Preven- tion, Chinese Center for Disease Control and Prevention, Beijing, 100871 China; 4 Division of HIV/AIDS and Sex-transmitted Virus Vaccines, Institute for Biological Product Control, National Institutes for Food and Drug Control (NIFDC) and WHO Collaborating Center for Standardization and Evaluation of Biologicals, Beijing, 102629 China; 5 Beijing Institute of Biotechnology, Beijing, 100871 China; 6 School of Basic Medicine, Fourth Military Medical University, Xi’an, 710032 China; 7 The Third People’s Hospital of Shenzhen, Shenzhen, 518112 China; 8 School of Medicine, Shanghai University, Shanghai, 200444 China

**Keywords:** Infection, Infectious diseases

## Abstract

The Omicron variants of SARS-CoV-2, primarily authenticated in November 2021 in South Africa, has initiated the 5th wave of global pandemics. Here, we systemically examined immunological and metabolic characteristics of Omicron variants infection. We found Omicron resisted to neutralizing antibody targeting receptor binding domain (RBD) of wildtype SARS-CoV-2. Omicron could hardly be neutralized by sera of Corona Virus Disease 2019 (COVID-19) convalescents infected with the Delta variant. Through mass spectrometry on MHC-bound peptidomes, we found that the spike protein of the Omicron variants could generate additional CD8 + T cell epitopes, compared with Delta. These epitopes could induce robust CD8 + T cell responses. Moreover, we found booster vaccination increased the cross-memory CD8 + T cell responses against Omicron. Metabolic regulome analysis of Omicron-specific T cell showed a metabolic profile that promoted the response of memory T cells. Consistently, a greater fraction of memory CD8 + T cells existed in Omicron stimulated peripheral blood mononuclear cells (PBMCs). In addition, CD147 was also a receptor for the Omicron variants, and CD147 antibody inhibited infection of Omicron. CD147-mediated Omicron infection in a human CD147 transgenic mouse model induced exudative alveolar pneumonia. Taken together, our data suggested that vaccination booster and receptor blocking antibody are two effective strategies against Omicron.

## Introduction

The Omicron variants of SARS-CoV-2, originally detected in Southern Africa have spread globally at a significantly higher rate than the Delta variant (www.who.int). About 32 mutations were found on the spike of Omicron, and most of them located in the N-terminal domain and the receptor binding domain (RBD). These mutation may change the viral fitness and allow immune evasion to neutralizing antibodies.^1–3^ Recently, several studies have substantiated that Omicron escapes the neutralizing antibodies and serum antibodies from convalescent patients or vaccinees.^4–7^ Ho and colleagues reported that compared to wild-type SARS-CoV-2, the ability of serum from vaccinated individuals to neutralize Omicron was decreased by 6.0-fold.^4^ And four spike mutations (S371L, N440K, G446S and Q493R) confer antibody resistance of Omicron.^4^ Given that SARS-CoV-2 is constantly mutating, it is necessary to develop new treatment strategies for Omicron and other emerging variants.

Thus far, vaccination is considered to be the main way to prevent COVID-19. Because of the decreased neutralizing capacity in neutralizing antibody caused by the vaccine, scientists focused on the antigen specific T cells induced by vaccination, especially CD8 + T cells. Virus specific CD8 + T cells can suppress viral replication and limit pathogenicity by eliminating infected cells.^8^ Studies have shown that CD8 + T cells immune response is critical in alleviating COVID-19 symptoms and triggering long-term immune protection. Clonal expansion of CD8 + T cells was observed in the lung tissues of patients with COVID-19,^9^ and in the peripheral blood of convalescent individuals.^10^ Expansion of CD8 + T cells was demonstrated to be related with milder disease severity and better viral clearance.^11^ Thus, CD8 + T cells which are specific to virus can be the main force for virus clearance, but its attack ability may be impaired by the continuous mutation of SARS-CoV-2, just like the neutralizing antibody.

Regarding the above speculation, the recognition ability of SARS-CoV-2 specific CD8 + T cells to the SARS-CoV-2 variant has been evaluated.^12–14^ As previously reported, spike-specific T-cells, induced by both mRNA-based and adenovirus-based vaccines, could recognize the spike proteins of SARS-CoV-2.^14–16^ Similar results were applied to the Omicron variants,^13,17,18^ indicating that although the multiple mutations in spike protein of the Omicron variants may interfere with T cell epitopes,^19^ this negative effect may be significantly weaker than that on neutralizing antibody epitopes. However, most of the these studies use peptide “megapools” containing 15-mer peptides overlapping by 10 amino acids as T cell epitopes. Although these peptides had been predicted to bind to MHC class I (MHC-I), they may not represent authentic MHC-I-binding peptides, which were shown to be 8–10 amino acids.^20^ Therefore, a formal test of antigen-specific T cells response to Delta- and Omicron-derived peptide epitopes is required to examine cross memory response of CD8 + T cells in convalescent individuals and vaccinees.

Here, we focus on whether T cell responses stimulated by vaccination (Sinovac-CoronaVac) cross-recognize Delta and Omicron variants using MHC-expressing artificial antigen-presentation cells (aAPCs). We use these aAPCs to generate an unbiased MHC-I immunopeptidome. Moreover, the function of antigen specific T cells must be fueled by specific metabolic programs that support biogenesis, migration and rapid cell expansion. However, metabolic state of Omicron-specific T cell had not been tested. Thus, we systemically examined immunological and metabolic characteristics of Omicron variants infection. We found Omicron resisted to neutralizing antibody targeting SARS-CoV-2 RBD, and antibodies were detected in the sera of COVID-19 convalescent individuals infected with the Delta variant. However, Omicron could induce strong antigen-specific CD8 + T cell responses when PBMCs from Delta convalescent individuals and vaccinees were stimulated with Omicron-derived T cell epitopes. Metabolic regulome analysis of Omicron-specific T cell showed a metabolic profile that promotes the response of memory T cells. Consistently, a greater fraction of CD8 + memory T cells were found in Omicron stimulated PBMCs. In addition, we found receptor blocking using CD147 antibodies could effectively blunt cellular entry of the Omicron variant. Taken together, our data suggested that vaccination booster and receptor blocking antibody are two effective strategies against Omicron.

## Results

### Omicron escaped the attack of neutralizing antibodies

**Fig. 1:**
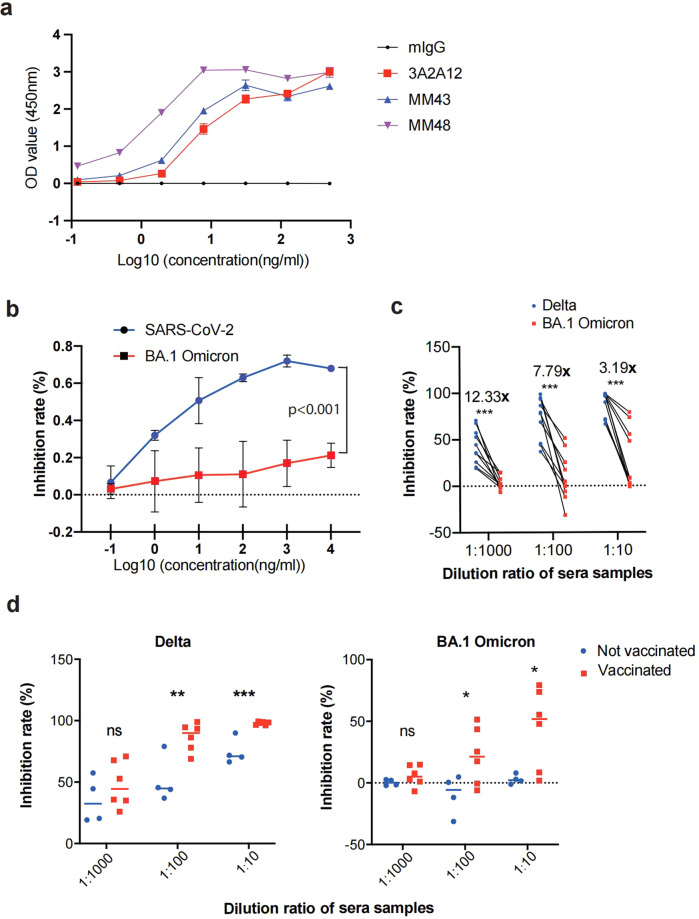
Immune escape by the BA.1 Omicron variant. **a** The ELISA assay shows the binding ability of 3A2A12, MM43, MM48 and mouse IgG to SARS-CoV-2 RBD (*n* = 4). **b** The neutralizing ability of 3A2A12 for SARS-CoV-2 and BA.1 Omicron pseudoviruses (*n* = 3), *p* < 0.001, determined by two-way ANOVA. **c** Inhibition of Delta and BA.1 Omicron pseudoviruses by convalescent patient sera (*n* = 10). **d** Inhibition of Delta (left) and BA.1 Omicron (right) pseudoviruses by COVID-19 convalescent patient sera in vaccinees (*n* = 6) and non-vaccinated group (*n* = 4). Data are represented as mean ± SEM, *p*-value was determined by the Wilcoxon rank sum test, (*p < 0.05, **p < 0.01, ***p < 0.001)

The receptor-binding domain (RBD) of the SARS-CoV-2 spike (S) protein plays a major role in the binding of SARS-CoV-2 with human angiotensin-converting enzyme 2 (ACE2), thus being considered an ideal target for neutralization antibody and convalescent plasma therapies.^21^ We generated an RBD-specific monoclonal antibody through hybridoma technology (hereafter referred as 3A2A12). Enzyme-linked immunosorbent assay (ELISA) assays were performed to detect the interaction of antibodies and S protein. 3A2A12 showed a comparable binding ability to S protein when compared with commercial antibodies (Fig. 1a). Next, wild-type SARS-CoV-2 or BA.1 Omicron pseudovirus were used to infect VeroE6 cells for virus neutralization assays. 3A2A12 could effectively neutralize wild-type SARS-CoV-2 but not BA.1 Omicron (Fig. 1b).

In addition, we examined the neutralizing efficiency of sera in ten convalescent individuals infected with the Delta variant (Table S1), which were used to test the neutralization efficiency to both Delta and BA.1 Omicron pseudoviruses. We found that the sera had reduced neutralizing efficiency against the BA.1 Omicron pseudovirus, compared with Delta (Fig. 1c), indicating that BA.1 Omicron could escape neutralizing antibodies. However, we found that sera from vaccinated patients had better inhibition for Delta and BA.1 Omicron than sera from non-vaccinated patients (Fig. 1d), indicating vaccination promoted cross immune memory response against Delta and BA.1 Omicron.

### Booster vaccination promoted cross immune memory response against Delta and BA.1 Omicron

To find out if BA.1 Omicron evades the COVID-19 related CD8 + T cell immune response, we isolated PBMCs from ten convalescent individuals infected with the Delta variant, who possessed at least one of three HLA haplotypes (HLA-A*02:01, HLA-A*11:01 and HLA-A*24:02) (Table S1). Blood collection time since test positive for COVID-19 were comparable for vaccinated and non-vaccinated patients (Table S1, p = 0.7493 by student *t*-test). To examine antigen specific CD8 + T cell responses, we created aAPCs, which are Cos-7 cells transfected with HLA-A and different spike proteins (Supplementary Fig. 1). Over expression of HLA-A was confirmed by quantitative PCR (Supplementary Fig. 2a). Single clonal cells that stably expressed S proteins (Supplementary Fig. 2b) were FACS-sorted for subsequent T cell stimulation assays.

**Fig. 2:**
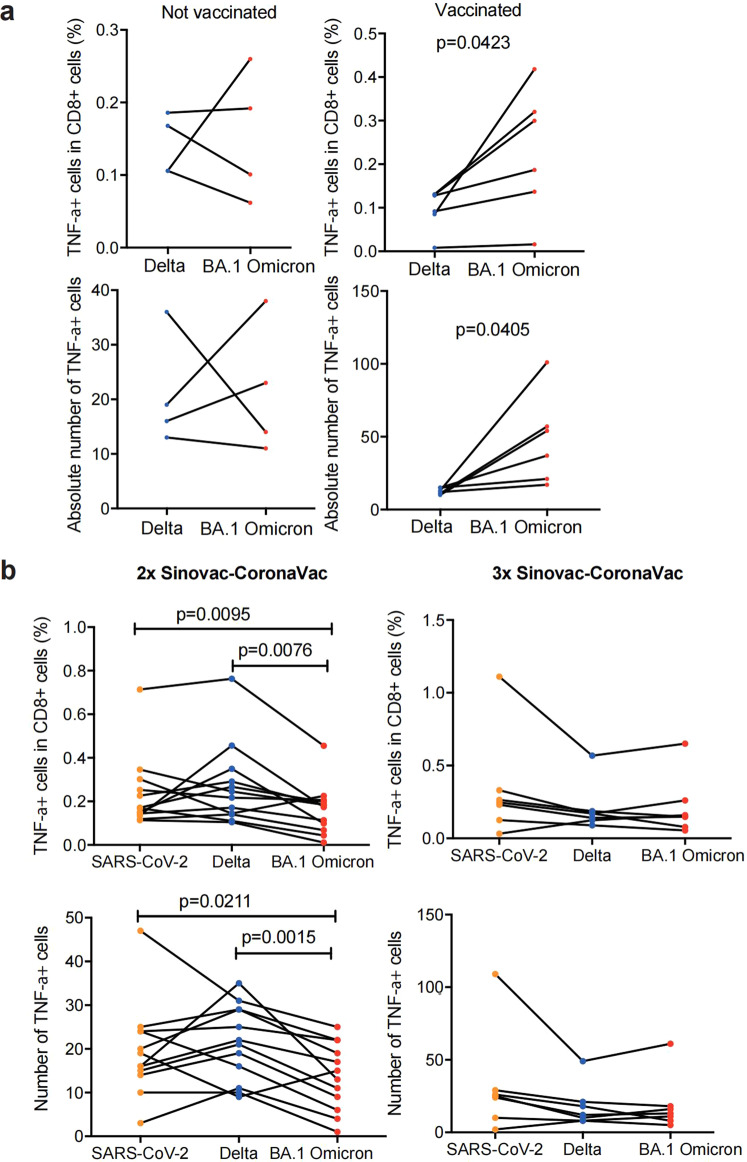
Immune response of Omicron to Delta-specific CD8 + T cells. **a** (Top) The percentage of TNF-α + cells in total CD8 + T cells in vaccinated convalescent individuals (*n* = 6) and convalescent individuals without vaccination (*n* = 4). (Bottom) The absolute number of TNF-α + cells in vaccinated convalescent individuals (*n* = 6) and convalescent individuals without vaccination (*n* = 4). **b** (Top) The percentage of TNF-α + cells in total CD8 + T cells in 2-doses vaccinees (*n* = 12) and 3-doses vaccinees (*n* = 7). (Bottom) The absolute number of TNF-α + cells in total CD8 + T cells in 2-doses vaccinees (*n* = 12) and 3-doses vaccinees (*n* = 7). Data are represented as mean ± SEM, *p*-value was determined by the wilcoxon rank sum test

To prove that the aAPCs can present viral-derived T cell epitopes, we transduced aAPCs with antigen from hepatitis B virus surface (HBsAg) and HLA-A*02:01. HLA-peptide complexes were purified and subjected to mass spectrometry (MS), which identified a hepatitis B virus (HBV)-derived peptide, confirming that the aAPCs could present viral antigens (Supplementary Fig. 3). Then, the aAPCs were transduced with genes encoding Delta or BA.1 Omicron spike proteins and then were used to stimulate T cells (Supplementary Fig. 1). The results showed that both vaccinees and non-vaccinated Delta convalescent individuals had CD8 + T cell responses against Delta and BA.1 Omicron (Fig. 2a). However, vaccinated patients showed a significantly higher CD8 + T cell response against BA.1 Omicron, based on intracellular staining of TNF-α, and the level of secreted IFN-γ (Fig. 2a, Supplementary Fig. 4). To examine Delta or BA.1 Omicron-derived peptide epitopes, the S protein of Delta or BA.1 Omicron transduced aAPCs were used to purify HLA-peptide complexes, which were then subjected to MS. Six BA.1 Omicron-derived peptide epitopes were found (Supplementary Fig. 5), while only three Delta-derived peptide epitopes were identified (Supplementary Fig. 6). Among these variants-specific peptide epitopes, EIDRLNEVAK,^22,23^ LNDLCFTNVYADSFVIR,^24^ NLREFVFKNIDGYFK have been previously described. In addition, we identified several peptide epitopes that were shared by the SARS-CoV-2, Delta and Omicron variants, which could be verified using in vitro T cell stimulation assays (Supplementary Fig. 7 and 8).

Previously infected subjects showed a stronger T cell response against spike protein, indicating that when being repeatedly exposed to antigens, the T cell responses with cross-reactivities may be potentially strengthened. Therefore, the effect of booster vaccination on the reactivity of antigen specific T cell was evaluated. We compared CD8 + T cell responses between 2-dose vaccinees and 3-dose vaccinees (with booster vaccination) (Table S2). These donors had no infection history of SARS-CoV-2.Two-dose vaccinees had significant reduction in TNF-α + CD8 + T cells (Fig. 2b), but not IFN-γ + CD8 + T cells (Supplementary Fig. 9), when their PBMCs were stimulated with BA.1 Omicron, compared with Delta (Fig. 2b), indicating that only 2-dose vaccination could not prevent T cell immune escape caused by the BA.1 Omicron variant. However, the majority of 3-dose vaccinees showed no significantly decreased T cell responses when their PBMCs were stimulated with BA.1 Omicron (Fig. 2b and Supplementary Fig. 9). These data showed that booster vaccination promoted cross immune memory response against Delta and BA.1 Omicron.

### BA.1 Omicron variant preferentially promoted the formation and maintenance of CD8+TEMs

**Fig. 3:**
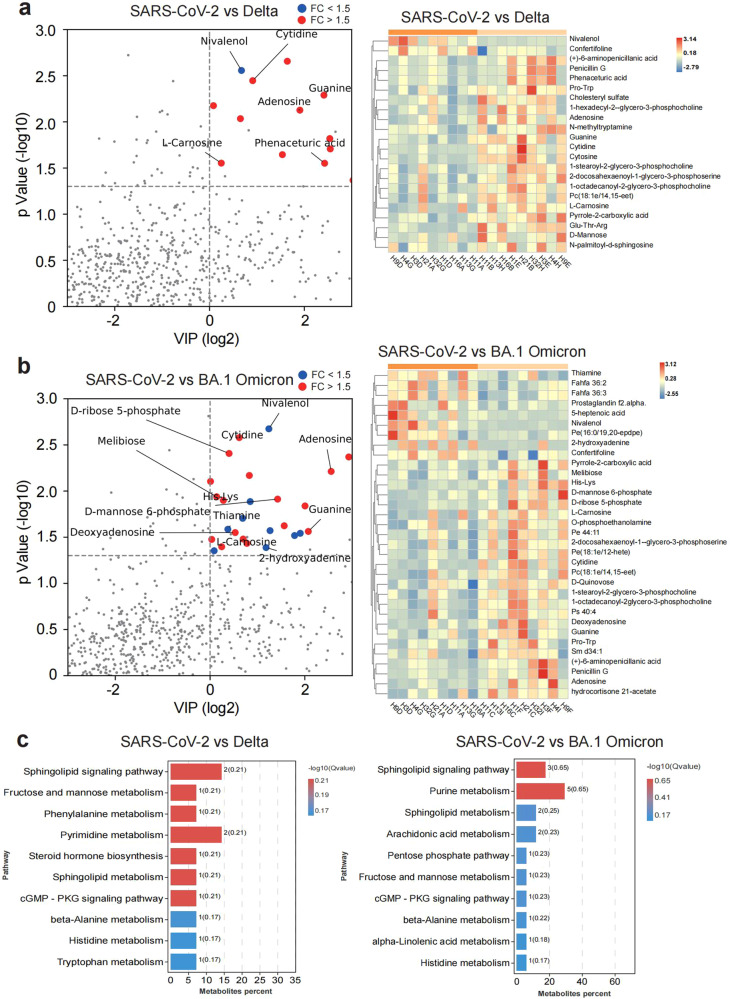
Metabolic profiling of SARS-CoV-2, Delta or Omicron-specific CD8 + T cells from 3-dose vaccinees. **a** The differential metabolic profiling of Delta vs SARS-CoV-2. **b** The differential metabolic profiling of BA.1 Omicron vs SARS-CoV-2. **c** KEGG analysis of difference metabolites of SARS-CoV-2 vs Delta, and SARS-CoV-2 vs Omicron, from 3-dose vaccinees. Data are represented as mean ± SEM

**Fig. 4:**
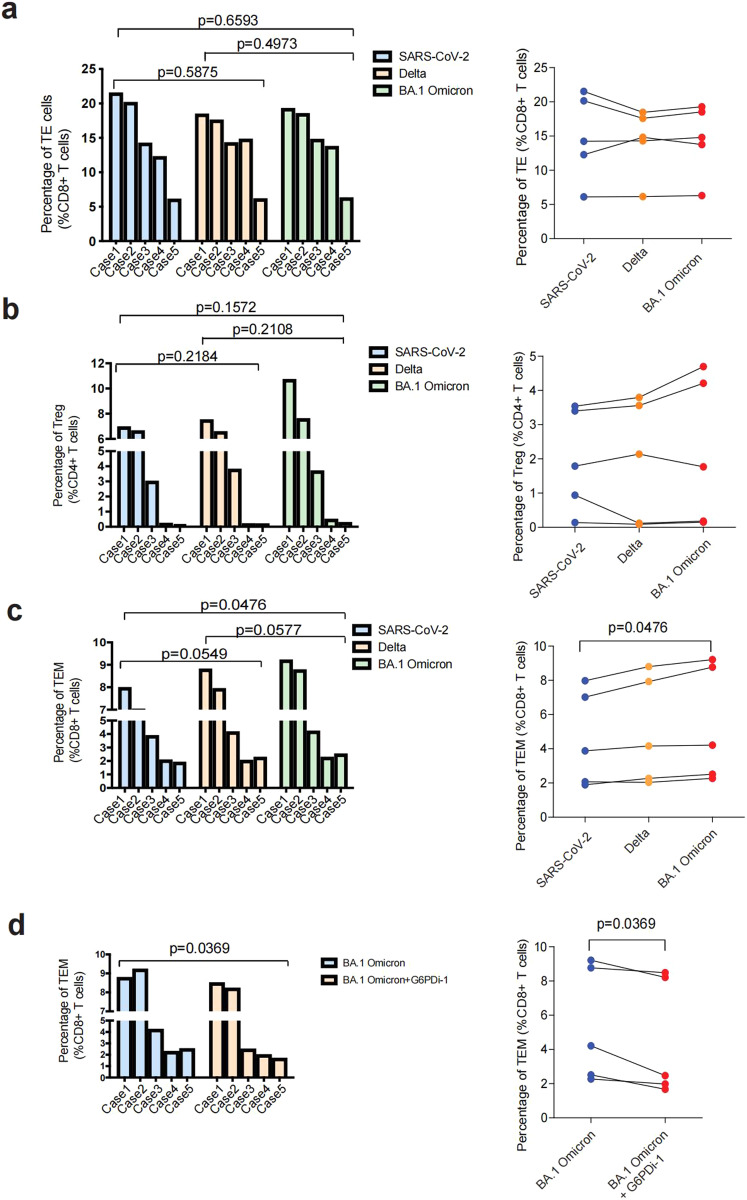
Difference in T cell differentiation after stimulated by SARS-CoV-2 variants. **a**–**c** Fraction of effector T cells (TE) (**a**), Treg cells (**b**), and effector memory T cells (TEM) (**c**) among SARS-CoV-2, Delta or Omicron-specific CD8 + T cells, respectively. **d** Fraction of TEMs in Omicron-specific CD8 + T cells before and after G6PDi-1 treatment. Data are represented as mean ± SEM, *p*-value was determined by two-way ANOVA

Following T cell receptor (TCR) triggering, T cells experience fierce metabolic reprogramming, increasing glucose use and glycolysis to fuel rapid cell expansion and effector function. To characterize metabolic regulomes of virus-specific T cells, PBMCs from 3-dose vaccinees were co-cultured with aAPCs presenting SARS-CoV-2, Delta and BA.1 Omicron, respectively, and subsequently subjected to metabolomics profiling. The metabolite profiles of T cells stimulated either by Delta or BA.1 Omicron variant were compared with those stimulated by SARS-CoV-2 (Fig. 3a, b). Upon activation, T cells undergo extensive clonal expansion, which is fueled mainly by glycolysis, as well as the pentose phosphate pathway (PPP). In addition to effector T cell (TE) responses, PPP had also been shown to promote the differentiation and maintenance of CD8 + memory T cells.^25^ We found both T cells stimulated by Delta and BA.1 Omicron variants were enriched for fructose and mannose pathway (Fig. 3b), suggesting that both Delta and BA.1 Omicron antigens increase the activation of memory T cell, compared to SARS-CoV-2 antigens. Notably, T cells stimulated by BA.1 Omicron antigen increased PPP activation, implying that BA.1 Omicron antigen may stimulate stronger activation and proliferation of memory T cells, or maintain the survival and production of memory T cells (Fig. 3b, c). To test this hypothesis, PBMCs from 3-dose vaccinees were stimulated with aAPCs presented with spike proteins of SARS-CoV-2, Delta and BA.1 Omicron, respectively. Post-stimulation cells were analyzed by flow cytometry. When compared with SARS-CoV-2, BA.1 Omicron stimulation did not significantly alter the frequency of TE and regulatory T cells (Tregs) (Fig. 4a, b). However, BA.1 Omicron stimulation produced larger fraction of CD8 + T effector memory cells (TEM) (Fig. 4c). This indicated that BA.1 Omicron stimulation preferentially promoted the survival and production of TEMs. The inhibitor of the PPP, G6PDi-1, could restore the fraction of TEM (Fig. 4d). We analyzed the extracellular acidification rate (ECAR) of antigen specific T cells triggered by antigens from SARS-CoV-2 variants. Glycolic pathway was activated in all groups, as shown by ECAR (Supplementary Fig. 10). These results indicated that T cells activated by SARS-CoV-2, Delta and BA.1 Omicron are of various activation states, in which BA.1 Omicron variant preferentially promoted formation and maintenance of CD8 + TEMs by increasing PPP.

### CD147 is a receptor for Omicron

CD147 was identified as a broad receptor mediating infection of SARS-CoV-2 and its variants Alpha, Beta, Gamma, and Delta.^26–28^ The silencing of CD147 showed similar inhibitory effect on infection of SARS-CoV-2 as Bromhexine hydrochloride, a specific inhibitor of TMPRSS2 (Supplementary Fig. 11). We tested if CD147 is a receptor for the Omicron variant. The affinity constant (K_D_) between Omicron RBD and CD147 was 5.24 × 10^−8^ M (Supplementary Fig. 12a), which was higher than that between CD147 and RBD of virus wildtype and its other variants.^26–28^ Then, CD147^-/-^VeroE6 cells were infected with the Omicron authentic virus. Loss of CD147 not only reduced SARS-CoV-2 infection, but also significantly reduced cellular entry of BA.1 and BA.2 variants (Supplementary Fig. 12b), with reduced infection rates of 72.9%, 76.0% and 55.0%, respectively, indicating CD147 is still an entry receptor for Omicron variants. Meplazumab, a humanized CD147 antibody that inhibits SARS-CoV-2 infection of host cells, has shown clinical benefits for COVID-19 patients.^27^ We then blocked infection of authentic SARS-CoV-2, BA.1 Omicron and BA.2 Omicron using meplazumab, which displayed that with the increase of concentration, inhibition rate of meplazumab gradually increased. Using 60 μg/ml of meplazumab, the inhibition rates to SARS-CoV-2, BA.1 Omicron and BA.2 Omicron were 56.2%, 60.7% and 41.0%, respectively (Supplementary Fig. 12c). The proliferation of VeroE6 cells was not affected by Meplazumab (Supplementary Fig. 12d). Thus, CD147 serves as a receptor for Omicron variants, and antibody targeting CD147 can effectively inhibit Omicron infection.

**Fig. 5:**
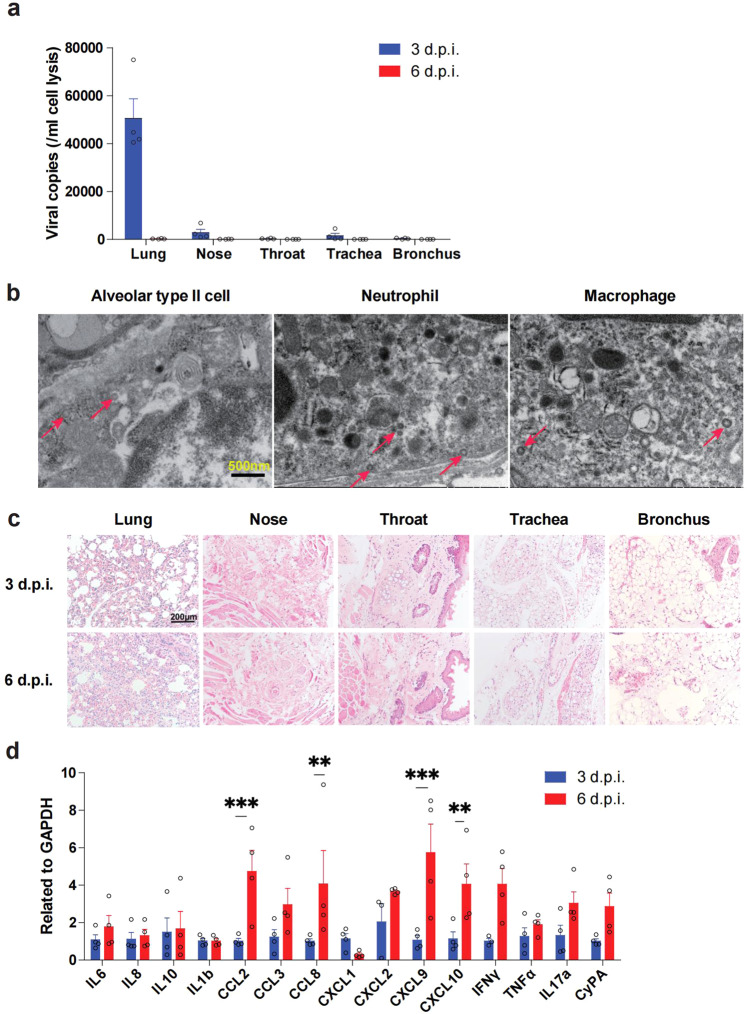
Human CD147 transgenic mouse model with BA.1 Omicron infection exhibited exudative alveolar inflammation. Inoculation mice via the intranasal at 3 × 10^5^ TCID_50_ of BA.1 Omicron, and sample collected at 3 and 6 d.p.i. **a** RT-qPCR for viral RNA levels in the lung, nose, throat, trachea, and bronchus (*n* = 4). **b** Virions in the alveolar type II cells, neutrophils and macrophages in lung tissues of human CD147 transgenic mice (at 3 d.p.i.) were detected by electron microscopy (scale bars, 500 nm). **c** H&E staining of tissues sections from infected human CD147 transgenic mice (scale bars, 200 μm). **d** Gene expression of cytokines and chemokines in lung homogenates determined by RT-qPCR (*n* = 4). Data are represented as mean ± SEM, *p*-values were determined by two-tailed student *t*-test, **p < 0.01, ***p < 0.001

To prove that CD147 is a receptor for BA.1 Omicron in vivo, we intranasally infected human CD147 (hCD147) transgenic mice with 3 × 10^5^ TCID_50_ of BA.1 Omicron variant. At 3 days post infection (d.p.i.), viral RNA at high levels was detected in lung tissues, and less viral RNA was found in upper respiratory tract tissues (nose, throat, trachea and bronchus) (Fig. 5a). The viral load dropped significantly in the lung tissue at 6 d.p.i. in comparison to 3 d.p.i. (Fig. 5a). Electron microscopy revealed the presence of virions in the alveolar type II cells, neutrophils and macrophages at 3 d.p.i. (Fig. 5b). Histologically, there was little damage to the upper respiratory tract organs after Omicron infection, while lung tissues exhibited exudative alveolar pneumonia, with the most severe signs at 6 d.p.i., featured by serofluid exudation, inflammatory cell infiltration, hemorrhage and pulmonary consolidation (Fig. 5c). This result was confirmed by multiplex immunofluorescence staining for macrophages, neutrophils, NK cells and CD4 + T cells in IgG and meplazumab group at 3 d.p.i. (Supplementary Fig. 13) and 6 d.p.i. (Supplementary Fig. 14). In addition, RT-qPCR (Fig. 5d) results showed that massive cytokines and chemokines increased at 6 d.p.i.. ELISA, performed to measure the serum level of cytokines and chemokines (IL-8, CCL2, IL17a, TNFα, IFNγ and IL6), showed the similar results (Supplementary Fig. 15a). These results indicate that the lung was still the primary target organ for BA.1 Omicron, mainly with exudative alveolar pneumonia.

**Fig. 6:**
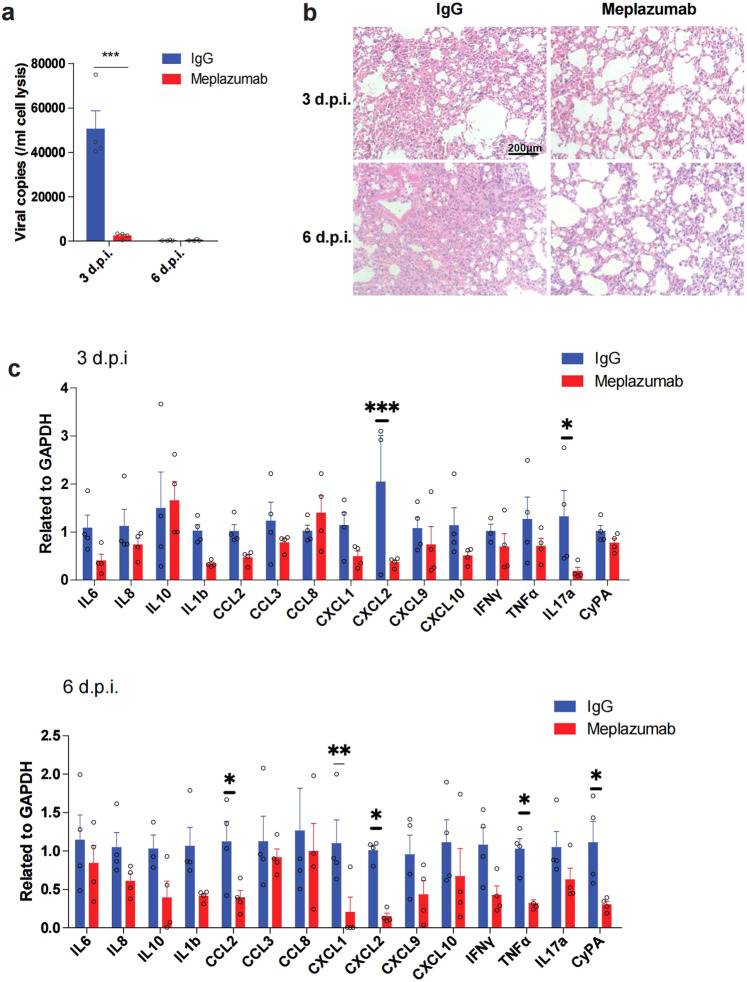
CD147 is a target for the treatment of COVID-19 caused by Omicron. Human CD147 transgenic mice were intranasally inoculated with 3 × 10^5^ TCID_50_ Omicron and treated with meplazumab the next day. **a** RT-qPCR for viral RNA levels in lung tissues at 3 and 6 d.p.i. (*n* = 4). **b** H&E staining of lung tissue sections from the IgG and meplazumab groups at 3 and 6 d.p.i. (scale bars, 200 μm). **c** Gene expression of cytokines and chemokines in lung homogenates determined by RT-qPCR, compared with the corresponding IgG controls at 3 d.p.i. (top) and 6 d.p.i. (bottom) (*n* = 4). *Gapdh* was used as a reference gene. Data are represented as mean ± SEM, *p*-values were determined by two-tailed student *t*-test, *p < 0.05, **p < 0.01, ***p < 0.001

Since human CD147 transgenic mice, when inoculated with authentic SARS-CoV-2, showed similar inflammatory manifestations to those of COVID-19 patients, the model is suitable for drug efficacy studies. Meplazumab has been evaluated in this model for treating pulmonary inflammation caused by wild-type SARS-CoV-2, alpha and beta variants^28^. However, its efficacy on the Omicron variants has not been evaluated. To further prove that meplazumab could block infection of Omicron, we treated Omicron-infected human CD147 transgenic mice with meplazumab. Meplazumab effectively reduced the virus load at 3 d.p.i. (Fig. 6a). Strikingly, at 6 d.p.i., the pneumonia was obviously alleviated after meplazumab treatment (Fig. 6b, Supplementary Fig. 15b). Moreover, the inhibition rates of the majority of cytokines, including IL-8, IL-10, IL-1β, CCL2, CCL8, CXCL1, CXCL2, CXCL9, CXCL10, TNF-α and CyPA increased at 6 d.p.i. when in comparison to 3 d.p.i. (Fig. 6c, Supplementary Fig. 15a). Thus, CD147 is a favorable target for the treatment of Omicron-caused COVID-19.

## Discussion

The Omicron variants struck global fear upon its detection in South Africa. The principal concerns are its high transmissibility, high rates of reinfection, and its high mutation load that may cause immune escape.^29^ Indeed, in our study, neutralizing antibody against RBD and sera from convalescents infected with Delta variant showed considerable reduction of neutralizing ability targeting Omicron, consistent with other studies.^4–7^ Except for antibodies, effector T cell and memory T cell responses can be stimulated by the infection and/or vaccination of SARS-CoV-2. The BA.1 Omicron consists of 15 RBD mutations, while the Delta variant has only two mutating sites in RBD. This has captured attention as to whether the effectiveness of current COVID-19 vaccines and neutralizing antibodies might be impaired.^30,31^ However, additional mutations in RBD may create additional T cell epitopes that can be presented by HLA. Indeed, we found that increased epitope peptides derived from Omicron could trigger enhanced the response of CD8 + T cells. Due to this mechanism, we found that overall magnitude of virus-specific CD8 + T cell responses toward spike protein of Omicron was robust. Moreover, we found booster vaccination increases the cross-memory response against Omicron. Therefore, although Omicron can escape neutralizing antibodies, it can still be mitigated by cross-reactive response of memory T cells. This may be one of the reasons that Omicron causes mild COVID-19 symptoms.^32^

Activated CD4 + and CD8 + T cells are activited and differentiated into terminal effector T cells or memory T cells upon acute infection, including TEM and central memory (TCM) cells. In a metabolic perspective, long-lived memory T cells favors the oxidative phosphorylation (OXPHOS) pathway over aerobic glycolysis, TEM cells rely mostly on aerobic glycolysis.^33^ Metabolic alterations in T cells were observed in SARS-CoV-2 infection relative to other pulmonary infectious diseases. Compared with influenza- or respiratory syncytial virus-specific cells, the expression of glycolysis-related genes were found to be decreased in SARS-CoV-2-responsive CD8 + T cells, which was associated with reduced CD8 + T cell polyfunctionality.^34^ In addition, metabolic reprogramming, induced by activation, is damaged in CD8 + T cells from COVID-19 patients.^35^ Specifically, in vitro–activated T cells from patients with COVID-19 displayed the decrease of glycolytic capacity and reserve, coupled with the relatively low activated mTOR signals. Here, our metabolic regulome analysis of Omicron-specific CD8 + T cell had a PPP-centric metabolic profile that promotes the response of memory T cells, in comparison to the Delta-specific CD8 + T cells. This phenotype is able to be reversed by G6PDi-1, an inhibitor of PPP pathway, indicating that Omicron-derived antigens can preferentially stimulate glycolysis pathways, which is required for TEM cells.

Although attempts to develop broad neutralizing antibodies had been made,^31^ a more generalized method to block viral entry is receptor blocking. Recently, CD147 was identified as a receptor mediating the entry of SARS-CoV-2 and its variants into host cells.^26–28^ In this work, virus inhibition experiments were performed using CD147 antibody (meplazumab), which showed that low concentration of meplazumab effectively suppressed the infection of Omicron, and further mitigated cytokine release storm. Therefore, meplazumab is a general blocker of SARS-CoV-2 VOCs, a favorable broad and effective drug treating COVID-19.

In summary, these findings strengthen our understanding about the immunological, pathological and metabolic mechanisms that may explain clinical symptoms of Omicron, and indicate that vaccine boosters can effectively protect hosts from the infection with SARS-CoV-2 variants. Our data thereby provide evidence for the continuous evaluation of how future viral mutation may escape antibody and T cell immune responses, which can justify the development of universal receptor blocking drugs.

## Materials and methods

### Virus, pseudovirus and cell lines

The SARS-CoV-2, BA.1 and BA.2 Omicron strains used for in vitro experiments were obtained from National Institute for Viral Disease Control and Prevention. The SARS-CoV-2 and Omicron pseudovirus expressing luciferase were obtained from the Institute for Biological Product Control, National Institutes for Food and Drug Control (Beijing, China). Generation of pseudovirus was described previously.^36–38^

### Generation of neutralizing antibody 3A2A12 targeting SARS-CoV-2 RBD

Balb/c mice (female, 8–12 weeks) were used to produce the neutralizing antibody targeting SARS-CoV-2 RBD. The antigen (SARS-CoV-2 Spike RBD-His Recombinant Protein, 40592-V08H, Sinobiological, 70 μg) was injected subcutaneously into the neck and back of mice after mixing with equal volume of Freund’s complete adjuvant. Two weeks later, repeat the antigen injection as above. This step was repeated once. Subsequently, 70 μg of antigen was injected by intraperitoneal injection and antibody titers were measured by ELISA assay. The last booster was given 3 days prior to cells fusion with 100 μg of antigen by intraperitoneal injection. Sp2/0 cells were used for fusion with spleen cells using PEG1500. After screening by HAT and HT medium, stable expression cell lines were finally obtained by multiple cloning culture and ELISA detection. For purification, the supernatant of monoclonal cells was collected for centrifugation at 8000 rpm for 30 mins and filtration using 0.2 µm strainer, and then the AKTA Purifier system was used for chromatography (Mabselect Xtra 25 ml, 17526907, Cytiva), the binding buffer and elution buffer were 0.02 M PBS buffer (pH7.3) and 0.05 M Glycine-hydrochloric acid buffer (pH2.45), respectively. Finally, the eluent was concentrated using 30 kD centrifugation ultrafiltration tube and stored at −80 °C.

### Pseudovirus neutralization assay

Neutralization was measured by Luciferase Reporter Assay System (Promega, E1960). SARS-CoV-2 pseudovirus was incubated with serum samples or 3A2A12 (an RBD-specific monoclonal antibody) in serial dilutions for 1 h at 37 °C. Then, the mixed samples were added to VeroE6 cells. After 24 h incubation at 37 °C with 5% CO_2_, the cells were washed with PBS and lysed using a 50 μl passive lysis buffer for 20 mins. Then, 100 μl luciferase assay reagent II was transferred to each well, and luciferase signals were obtained from a luminometer (E5311, Promega) or Cytation 1 (Gain 135).

### In vitro pseudovirus infection test

The pseudovirus of SARS-CoV-2 and Omicron were piped to VeroE6 cells at a 50% Tissue Culture Infectious Dose (TCID_50_) of 1300 with 60 μg/ml of meplazumab (Jiangsu Pacific Meinuoke Biopharmceutical Co. Ltd.). The control group was performed with human IgG. Dual-Luciferase Reporter Assay System (E1960, Promega) was adopted to detect the pseudovirus infection.

### Plasma and PBMCs samples

Plasma of convalescent COVID-19 subjects (Table S1) were collected from the Third People’s Hospital of Shenzhen. Plasma of vaccinees were obtained from Xijing Hospital (Table S2). This study obtained the approval from the ethics committees of the Third People’s Hospital of Shenzhen and Xijing Hospital, respectively.

### Transfection of Cos-7 cells lines expressing HLA-A*02:01, HLA-A*11:01 and HLA-A*24:02

The stable transfectant Cos-7 cell expressing HLA-A*02:01, HLA-A*11:01 and HLA-A*24:02 were established by Shanghai Genbase Biotechnology. Transfection of these stable transfectant in Cos-7 cells was conducted with Lipofectamine 2000, which were established with the Delta or Omicron spike genes.

### Ligandome LC-MS/MS analysis

For HLA class Ι Hepatitis B virus peptides: The enriched HLA class Ι Hepatitis B virus peptides samples after fractional processing was resolved in buffer A, injecting of each into a nano Elute (UHPLC) for analysis, respectively. Buffer A is 0.1% FA in ultrapure water and Buffer B is 0.1% FA in acetonitrile. A flow rate of 300 nL/min was used to separated all peptides, with a gradient of 2–22% buffer B for 45 min, 22–80% buffer B for 15 min, while a timsTOF Pro2 mass spectrometer (Bruker DaltonicsTM) was coupled online with the nanoElute. Data acquisition method was set with a full scan MS from m/z 300 to 1500 with the resolution 60,000, and the ion mobility range (1/K0) was 0.75 − 1.3 Vs/cm^2^. The precursors were fragmented in CID with collision energy from 20ev–59ev, then MS/MS scanned.

For HLA class Ι Delta/Omicron BA.1 peptides: The tryptic peptides were dissolved in 0.1% formic acid (solvent A), directly loaded onto a home-made reversed-phase analytical column (15-cm length, 75 μm i.d.). The gradient was comprised of an increase from 6 to 23% solvent B (0.1% formic acid in 98% acetonitrile) over 16 min, 23 to 35% in 8 min and climbing to 80% in 3 min then holding at 80% for the last 3 min, all at a constant flow rate of 400 nl/min on an EASY-nLC 1000 UPLC system. The peptides were subjected to NSI source followed by tandem mass spectrometry (MS/MS) in Q ExactiveTM Plus (Thermo Fisher Scientific) coupled online to the UPLC. The electrospray voltage applied was 2.0 kV. The m/z scan range was 350 to 1800 for full scan, and intact peptides were detected in the Orbitrap at a resolution of 70,000. Peptides were then selected for MS/MS using NCE setting as 28 and the fragments were detected in the Orbitrap at a resolution of 17,500. A data-dependent procedure that alternated between one MS scan followed by 20 MS/MS scans with 20.0 s dynamic exclusion.

### Ligandome data analysis

For HLA class Ι Hepatitis B virus peptides: TimsToF Data files were searched using PEAKS Database Search in PEAKS Studio 10.6 against the Hepatitis B virus protein sequence database (Uniprot ID No.Q2F515). The enzyme and digest mode was set to none and unspecific with a precursor mass tolerance 20 ppm and fragment mass tolerance 0.05 Da. Methionine oxidation (+15.995 Da) was set as variable modifications. Peptide score filter was set to PSM 1% FDR.

For HLA class Ι Delta/Omicron BA.1 peptides: Sequest HT in Proteome Discoverer 2.4 (Thermo Fisher Scientific) was used to search the raw files. The search was set to no-enzyme (Unspecific) with a precursor mass tolerance 10 ppm and fragment mass tolerance 0.02 Da. Methionine oxidation (+15.995 Da), N-terminal Methionine Met-loss (−131.040 Da), Met-loss + Acetyl (−89.030 Da) and N-terminal acetylation (+42.011 Da) were set as variable modifications. General Validation Mode is Automatic (without control of peptide level error rate), and the identified peptides were filtered against using the Fixed Value PSM Validator. Peptide Confidence At Least set as Medium and Confidence Thresholds The Strict and Relaxed of Target FDR set to 0.01 and 0.05, respectively.

### Metabolomics

PBMCs from nine volunteers (3-dose vaccinees) were used in this study. Each PBMCs was stimulated with APCs presented with spike protein of SARS-CoV-2, Delta and Omicron, respectively. We collected these cells 1 day later for metabolic analysis. Detailed procedures are provided in the Supplementary Material.

### Infection of hCD147 mice with BA.1 Omicron

Mice were anesthetized and intranasally infected with BA.1 Omicron at a TCID_50_ of 3 × 10^5^. At 1 d.p.i., 4 mg/kg IgG or meplazumab was administered through the tail vein. The collected lung tissues were used for RNA extraction, and fixed with paraformaldehyde for H&E staining. The quantification of inflammatory conditions in H&E sections was calculated as follows: the inflammatory area/total area (%).

### Statistical analysis

Significant differences were analyzed by unpaired *t*-tests with a two-tailed distribution, Wilcoxon rank sum test and two-way ANOVA. ELISA data were analyzed using a parameter logistic curve. *p* < 0.05 was statistically significant. Statistical analyses were performed using GraphPad Prism, version 9.0.

## Supplementary information

Supplementary_Materials and data

## Data Availability

The datasets generated and analysed during the current study are available in the public repository (accession number: PXD037955). And all data will be available upon request to corresponding authors.
