# Supplementary material for: Immunological and metabolic characteristics of the Omicron variants infection
Source: Signal Transduct Target Ther. 2023 Jan 21;8:42. doi: 10.1038/s41392-022-01265-8 (PMC9860238; doi:10.1038/s41392-022-01265-8)
Supplement: Supplementary file 1 — Supplementary_Materials and data [file 41392_2022_1265_MOESM1_ESM.docx]

Supplementary Materials for

Immunological and Metabolic Characteristics of the Omicron Variants Infection

Correspondence to: Liang Chen (lchen1@shu.edu.cn), Huijie Bian (hjbian@fmmu.edu.cn), Ping Zhu [(zhuping@fmmu.edu.cn),](mailto:(zhuping@fmmu.edu.cn),) Hongzhou Lu [(luhongzhou.fudan.edu.com),](mailto:(Gengjie-jie@163.com),) Yingxia Liu (yingxialiu@hotmail.com)

**This PDF file includes:**

Materials and Methods

Figures. S1 to S15

Tables S1 to S2

**Materials and Methods**

**Virus, pseudovirus and cell lines**

The SARS-CoV-2, BA.1 and BA.2 Omicron strains used for in vitro experiments were obtained from National Institute for Viral Disease Control and Prevention. The SARS-CoV-2 and Omicron pseudovirus expressing luciferase were obtained from the Institute for Biological Product Control, National Institutes for Food and Drug Control (Beijing, China). Generation of pseudovirus was described previously ^35-37^. VeroE6 and H1299 cells were obtained from the Cell Bank of the Chinese Academy of Sciences (Shanghai, China). Cos-7 cell line was purchased from Genbase Biotechnology (Shanghai, China). All cell lines were authenticated using short tandem repeat DNA profiling at Beijing Microread Genetics Co., Ltd. (Beijing, China) and cultured at 37°C under 5% CO_2_ in Dulbecco’s modified Eagle’s medium (DMEM, Invitrogen) or RPMI 1640 medium supplemented with 10% fetal bovine serum (FBS, Life Technologies), 1% penicillin/streptomycin and 2% L-glutamine.

**Mice**

hCD147 mice were described previously ^27^. These mice were bred in a specific pathogen-free environment of the Chinese Academy of Medical Sciences. The animal experiments were approved by the People’s Republic of China Legislation Regarding the Use and Care of Laboratory (Animals ethics number 2021-NTSCMM-001).

**Enzyme-linked immuno sorbent assay (ELISA)**

ELISA assay was performed to identify the interaction of neutralizing antibodies and spike protein. The His-spike (RBD) protein was coated on microplate, and then incubated with different concentrations of 3A2A12, mIgG, MM43 (Sino Biological, 40591-MM43) and MM48 (Sino Biological, 40592-MM48) at 37°C for 1 hour. After washing with PBST, incubated with horseradish peroxidase HRP-labeled goat anti-mouse antibody (dilution ratio 1:6000) for 1 hour. After coloration, the OD value at 450 nm was measured with a full-wavelength microplate reader (Epoch, BioTek Instruments, Inc., USA).

**Flow cytometry analysis**

Flow cytometry was performed using BD Fortessa. For intracellular cytokine staining, cells were stimulated with 5 μg/ml brefeldin A (BioLegend) and 2 μM monensin (BioLegend) for 6 hours. For intracellular standing, cells were treated with a Fixation/Permeabilization solution kit from BD Cytofix/Cytoperm™ and stained with anti-cytokine antibodies. Fluorochrome-conjugated antibodies recognizing CD8, IFN-$\gamma$ and TNF-$\alpha$ were purchased from BioLegend.

**Production and purification of HLA class Ι peptides**

$2\times{10}^{7}$cells were harvested by centrifugation and washed three times with cold phosphate-buffered saline (PBS). Cells were lysed for 1.5 hours at 4°C in lysis buffer. The lysis buffer consisted of Pierce IP lysis buffer (Thermo Fischer Scientific, 0.025M Tris, 0.15M NaCl, 0.001M EDTA, 1% NP-40, 5% glycerol; pH7.4) supplemented with 1x EDTA-free Protease Inhibitor Cocktail (Roche Diagnostics), 50 μg/ml DNase Ι (Sigma-Aldrich), and 50 μg/ml RNase A (Sigma-Aldrich). Subsequently, the lysate was cleared by centrifugation for 1 hour at 18000g at 4°C. Protein concentration was determined with the BCA protein concentration assay kit (Beyotime). HLA class Ι complexes and peptide ligands were co-immunoprecipitated using W6/32 antibody coupled to AminoLink Plus Coupling Resin (Thermo Fischer Scientific) from whole-cell lysate. Incubation took place at 4 °C for approximately 16 hours. After co-immunoprecipitation, the resin was washed with cold PBS. HLA class Ι complexes and peptide ligands were subsequently eluted with 10% acetic acid. Peptide ligands were separated from HLA class Ι complexes using 10 kDa molecular weight cutoff filters (Millipore). The flow-through containing the HLA class Ι peptide ligands was dried by vacuum centrifugation.

**Surface plasmon resonance (SPR)**

The Omicron RBD of spike protein was obtained from GenScript (Nanjing, China). SPR analysis was performed by BIAcore 3000 system (BIAcore, USA). His-CD147 (produced by our laboratory) was fixed to the surface of CM5 sensor chips (GE Healthcare Bio-Sciences AB) by amino coupling kit (GE Healthcare, BR-1000-50). The interaction between CD147 and Omicron RBD was detected using Kinetic Analysis/Concentration Series/Direct Binding mode, and the flow rate was set to 15 μl/minute, both binding time and dissociation time were 3 minutes. The results were analyzed by BIA evaluation software to determine the affinity constant.

**Metabolomics**

We used PBMCs from 9 volunteers (3-dose vaccinees), each of whose PBMCs was stimulated with APCs presented with SARS-CoV-2, Delta, and Omicron, respectively. Cells were collected 1 days later for metabolic analysis. Detailed procedures are provided in the Supplementary Material. The culture medium from the cultured cells was removed using pipette. Then the cells were washed with PBS under 37 °C and the PBS was removed. 800 μl of cold methanol/acetonitrile (1:1, v/v) to remove the protein and extract the metabolites. The mixture was collected into a new centrifuge tube and centrifuged at 14000g for 5 min to collect the supernatant. The supernatant was dried in a vacuum centrifuge. For LC-MS analysis, the samples were re-dissolved in 100 μl acetonitrile/water (1:1, v/v) solvent. To monitor the stability and repeatability of instrument analysis, quality control (QC) samples were prepared by pooling 10 μl of each sample and analyzed together with the other samples. The QC samples were inserted regularly and analyzed in every 5 samples.

LC-MS/MS analyses were performed using an UHPLC system (1,290, Agilent Technologies) with a UPLC HSS T3 column (2.1 mm × 100 mm, 1.8 μm) coupled to a Q Exactive mass spectrometer (Orbitrap MS, Thermo). The mobile phase A was 0.1% formic acid in water for positive mode, and 5 mmol/l ammonium acetate in water for negative mode, and the mobile phase B was acetonitrile. The elution gradient was set as follows: 0 min, 1% B; 1 min, 1% B; 8 min, 99% B; 10 min, 99% B; 10.1 min, 1% B; 12 min, 1% B. The flow rate was 0.5 ml/min. The injection volume was 2 μL. The QE mass spectrometer was used due to its ability to acquire MS/MS spectra on an information-dependent basis (IDA) during an LC/MS experiment. In this mode, the acquisition software (Xcalibur 4.0.27, Thermo) continuously evaluates the full scan survey MS data as it collects and triggers the acquisition of MS/MS spectra depending on preselected criteria. ESI source conditions were set as follows: sheath gas flow rate was 45 Arb, aux gas flow rate was 15 Arb, capillary temperature was 320°C, full MS resolution was 70,000, MS/MS resolution was 17,500, collision energy was 20/40/60 eV in the NCE model, spray voltage was 3.8 kV (positive) or -3.1 kV (negative), respectively.

The raw data files generated by UHPLC-MS/MS were processed using the Compound Discoverer 3.1 (CD3.1, Thermo Fisher) to perform peak alignment, peak picking, and quantitation for each metabolite. The main parameters were set as follows: retention time tolerance, 0.2 mins; actual mass tolerance, 5ppm; signal intensity tolerance, 30%; signal/noise ratio, 3; and minimum intensity, 100,000. After that, peak intensities were normalized to the total spectral intensity. The normalized data was used to predict the molecular formula based on additive ions, molecular ion peaks and fragment ions. And then peaks were matched with the mzCloud (https://www.mzcloud.org/), mzVault and Mass List database to obtain the accurate qualitative and relative quantitative results. Statistical analyses were performed using the statistical software R (R version R-3.4.3), Python (Python 2.7.6 version) and CentOS (CentOS release 6.6). When data were not normally distributed, normal transformations were attempted using of area normalization method. The variance of metabolites across samples was calculated in positive and negative ion modes according to the LC-MS spectra. Metabolites showing a p<0.05 and VIP>1 were considered as deferentially expressed metabolites. For statistical analysis, principal component analysis, partial least squares discriminant analysis (PLS-DA), and orthogonal projection to latent structures discriminant analysis (OPLS-DA) were applied to compare groups using the R package models (http://www.r-project.org/).

**Generation of CD147^-/-^VeroE6 cell line**

CD147^-/-^VeroE6 cells were generated using the CRISPR/Cas9 system (GeneChem Co. Ltd).

**In vitro virus infection test**

The cells were cultured at 37°C under 5% CO_2_ overnight, the supernatant was discarded and 100 μl of medium (containing 15~120 μg/ml meplazumab) was added into the plates to incubate for 1 hour. The cell medium was then replaced by medium containing the SARS-CoV-2 and Omicron virus. After the cells were infected for 1 hour at 37°C, the virus supernatant was discarded, and the cells were washed twice with PBS. Finally, the cells were cultured with 2% FBS maintenance medium with meplazumab (15~120 μg/ml). At 48 hours after infection, viral RNA and cellular RNA were extracted together and detected by RT-qPCR.

**RNA extraction and real-time quantitative PCR analysis**

Total RNA was extracted using Total RNA Kit II (Omega Biotek) according to the manufacturer’s instructions. cDNA was synthesized from 1 μg of total RNA using an RNA reverse transcriptase kit (Takara). Real-time qPCR was performed using the ABI PRISM 7000 Sequence Detection System (Applied Biosystems), and SYBR Premix Ex Taq II (Takara) was used for amplification according to the manufacturer’s instructions. The cDNA inputs were standardized, and PCR was performed for 40 cycles. The primer sequences for SARS-CoV-2 N protein, cytokines and chemokines were described previously ^27^.

**Electron microscopy**

Electron microscopy was described previously ^25^.

**Cell viability analysis**

VeroE6 cells seeded in 96-well plates were incubated with different concentrations of meplazumab (0, 15, 30, 60, 120 μg/ml) at 37°C for 24 hours. Then, the supernatant was removed and 100 μl DMEM containing 10 μl CCK-8 reagent was added to each well. After incubating for 1 hour, the optical density (OD) value was detected at 450 nm using a microplate reader (BioTek Epoch).

**Protease-inhibitor treatment assay**

The serine protease inhibitor, Bromhexine hydrochloride (HY-B0372A, MedChemExpress), was used to perform pseudovirus infection assay. H1299 cells were treated with DMSO or bromhexine hydrochloride at concentrations of 25, 50, and 100 µM for 2 hours before pseudovirus transduction. After 24 hours incubation, the cells were lysed for detecting luciferase signal with Dual-Luciferase Reporter Assay System (E1960, Promega).

**Detection of IFN-**$\boldsymbol{\gamma}$ **producing cells by Elispot**

IFN-$\gamma$ Elispot was used to evaluate T cell responses. PBMCs were stimulated with 1 µg/ml SARS-CoV-2-derived peptide pool, in triplicate, in 3 × 10^5^ cells/well in RPMI-1640 medium containing 10% fetal calf serum, for 6 days at 37°C and 5% CO_2_. DMSO-stimulated cells served as negative controls. Subsequently, the number of IFN-$\gamma$-producing cells was measured using human IFN-$\gamma$ precoated Elispot kit (2110005). Spot numbers were counted using an Immunospot reader (version 3.0) and software (version 6.1) (CTL Immunospot S6 Ultra-V Analyzer, Bonn, Germany).

**Extracellular acidification rate assay (ECAR)**

The Cos-7-HLA-A*02:01 cells (1.5 × 10^4^/well) infected with SARS-CoV-2, Delta and Omicron spike gene lentivirus were seeded in 48-well plate, respectively. T cells (1.5 × 10^5^/well) from three healthy volunteers (HLA-A*02:01) who received three doses of SARS-CoV-2 vaccine were co-cultured with Cos-7-HLA-A*02:01 cells mentioned above for 24 hours. The cells in 48-well plate were filtered through a 75 μm filter, and the collected T cells were seeded in 96-well plate for Seahorse XFe96 Analyzer and let stand for 1 hour. Next, oligomycin (1 μM), 2-deoxy-glucose (50 mM) and glucose (10 mM) were added to determine ECAR value using Seahorse XF Glycolysis Stress Test Kit (103020-100, Agilent Technologies) through Seahorse XFe96 Analyzer. The data were analyzed using Wave software (Agilent Technologies).

Figure. S1.


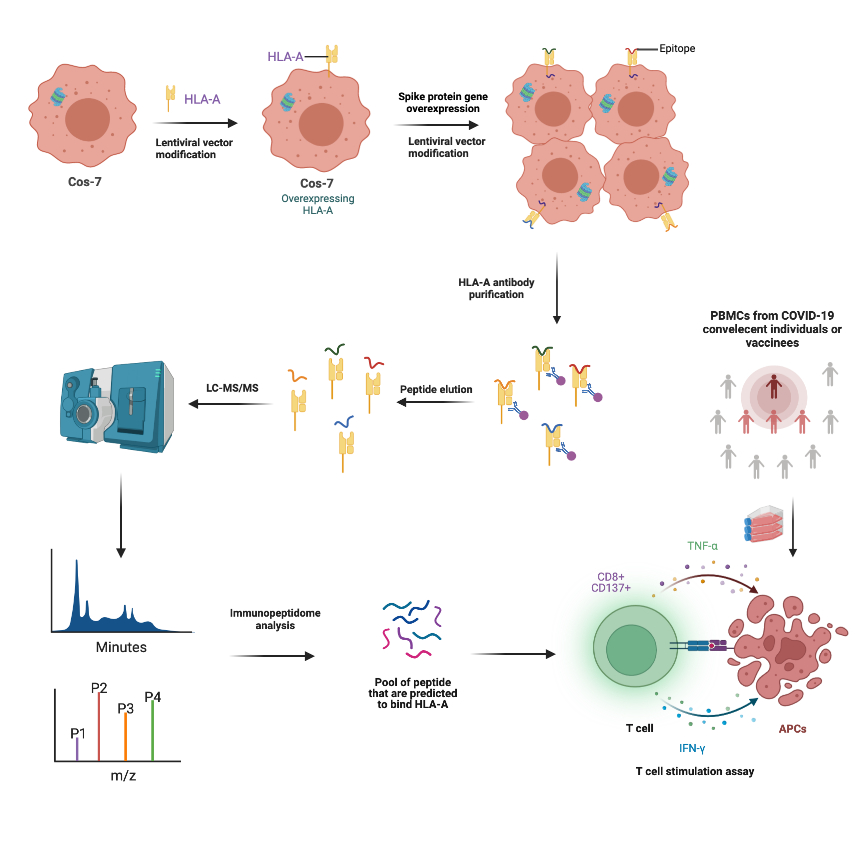


Figure. S1.

Workflow of LC-MS based Immunopeptidome and in vitro T cell stimulation assays. The aAPC was built on the Cos-7 cell line with stable expression of HLA-A*02:01, HLA-A*24:02 or HLA-A*11:01. Viral proteins were expressed, processed, and presented by aAPC; the peptide-MHC complex was used to stimulate PBMCs of human subjects.

Figure. S2.


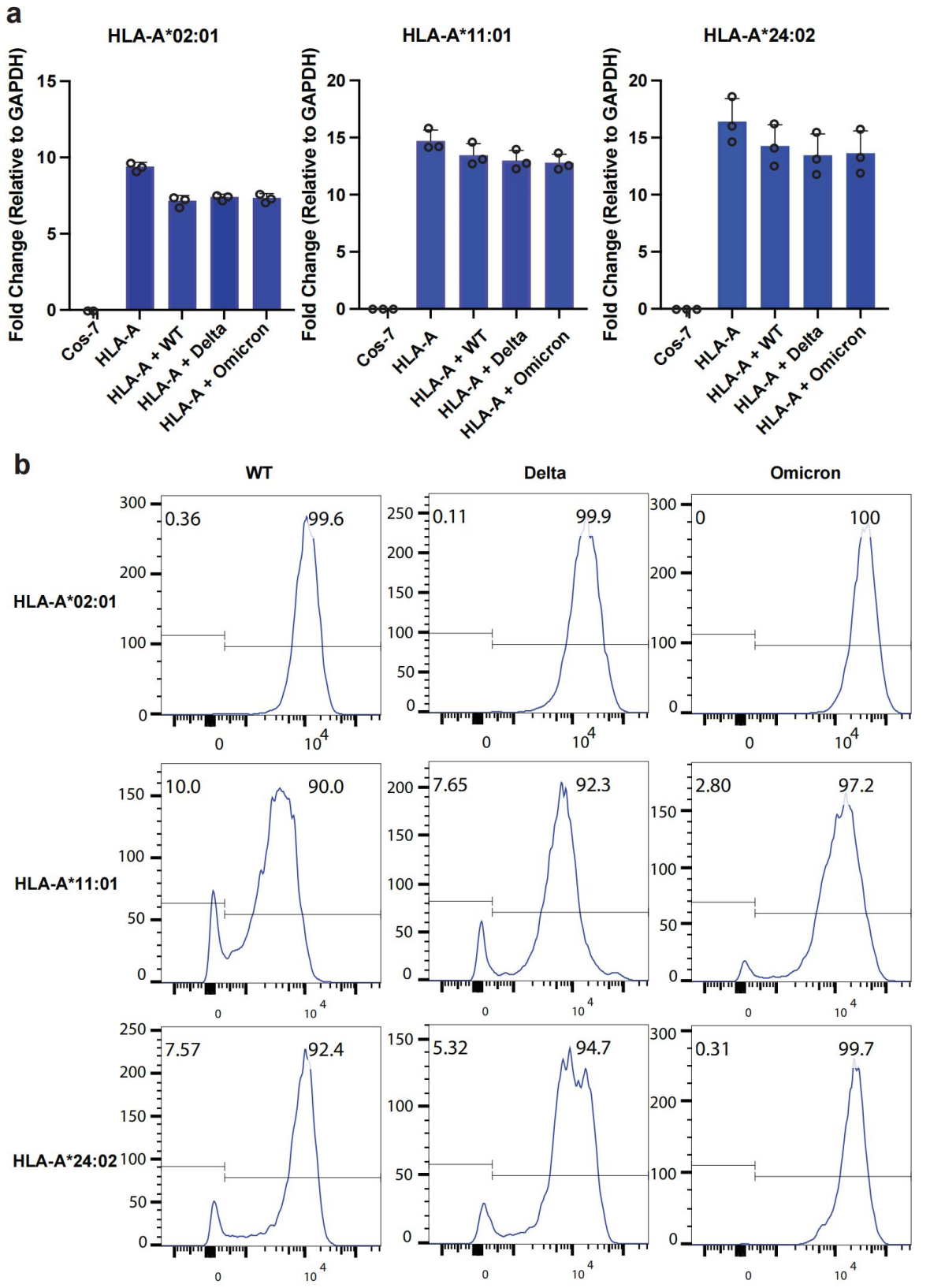


Figure. S2.

**a** Expression of HLA-A quantified by real-time PCR. **b** Flow cytometry of S protein of SARS-CoV-2, Delta, and Omicron variants. WT: SARS-CoV-2; Omicron: BA.1 Omicron. Data are represented as mean ± SEM.

Figure. S3.


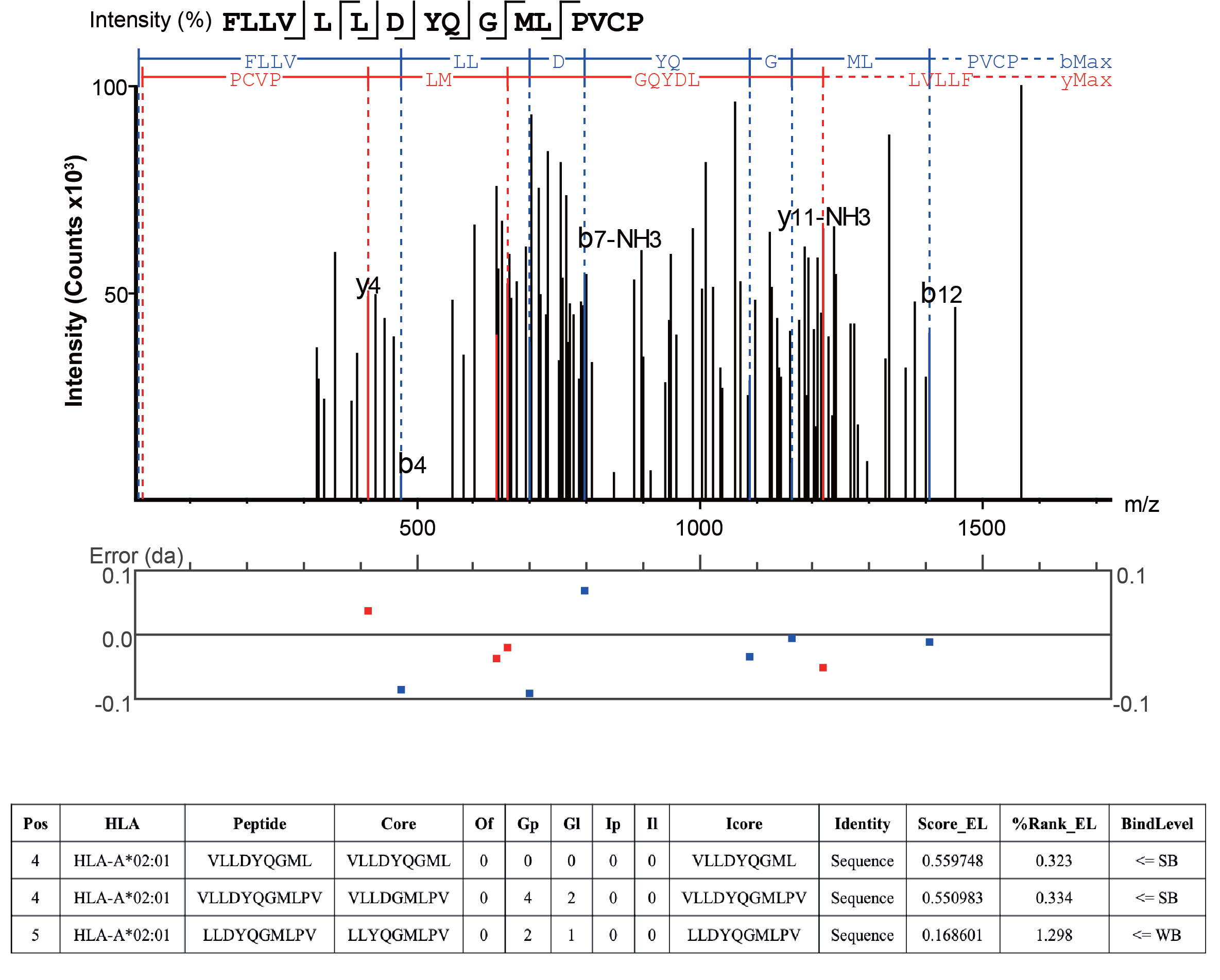


Figure. S3.

LC-MS/MS spectrum of the HBV peptide FLLVLLDYQGMLPVCP identified in Cos-7-HLA-A*02:01 cells. (Top) Spectrum images of the peptide were extracted from PEAKS Studio. (Bottom) Prediction of binding strength of FLLVLLDYQGMLPVCP to HLA alleles HLA-A*02:01 using NetMHCpan-4.1.

Figure. S4.


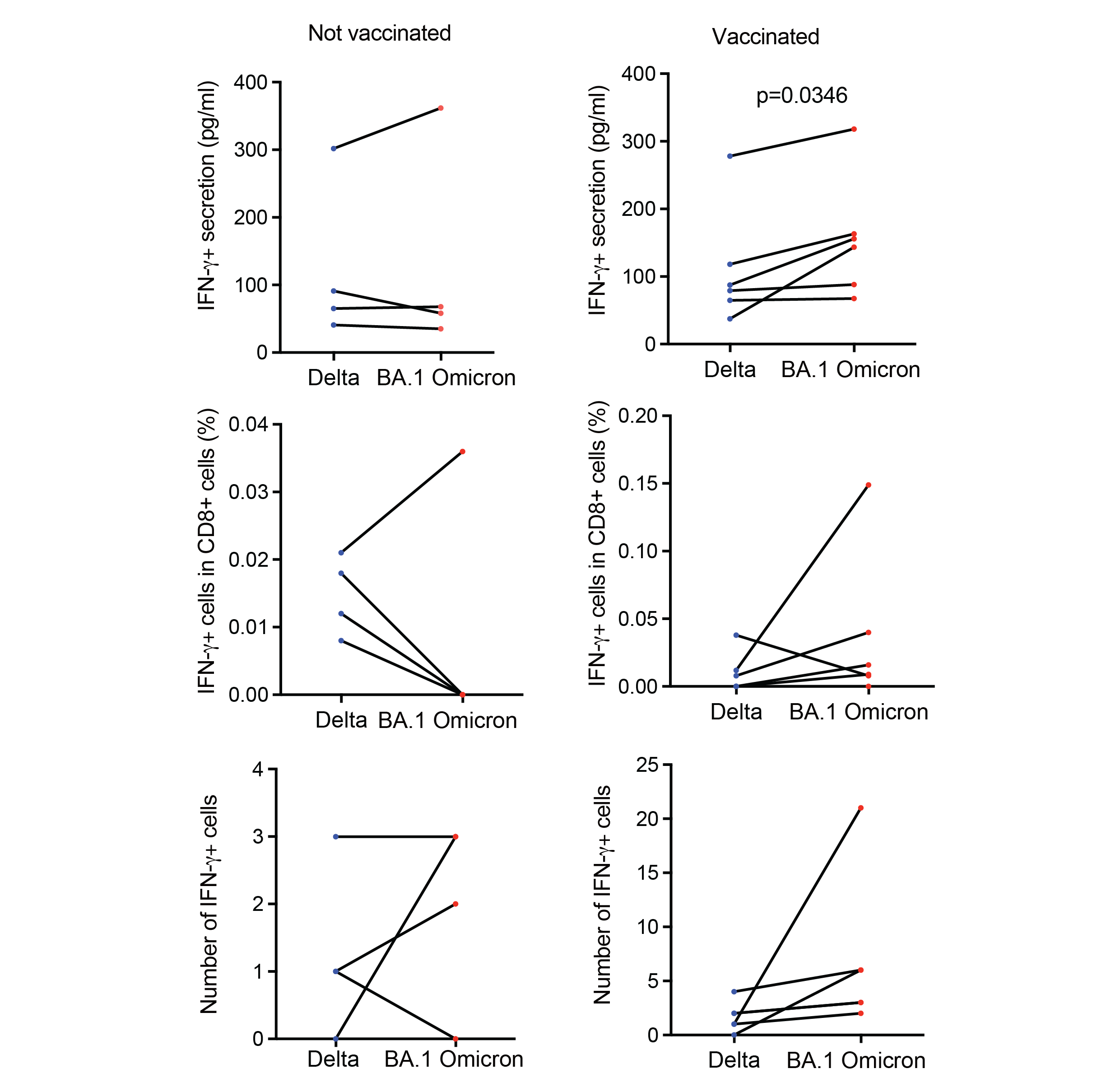


Figure. S4.

Immune response of Omicron to Delta-specific CD8+ T cells. (Top) Secretion of IFN-$\gamma$ quantified by ELISA. (Center) The percentage of IFN-$\gamma$ cells in total CD8+ T cells in vaccinated convalescent individuals (n=6) and convalescent individuals without vaccination (n=4). (Bottom) The absolute number of IFN-$\gamma$ cells in vaccinated convalescent individuals (n=6) and convalescent individuals without vaccination (n=4). p values were determined by the Wilcoxon rank sum test.

Figure. S5.


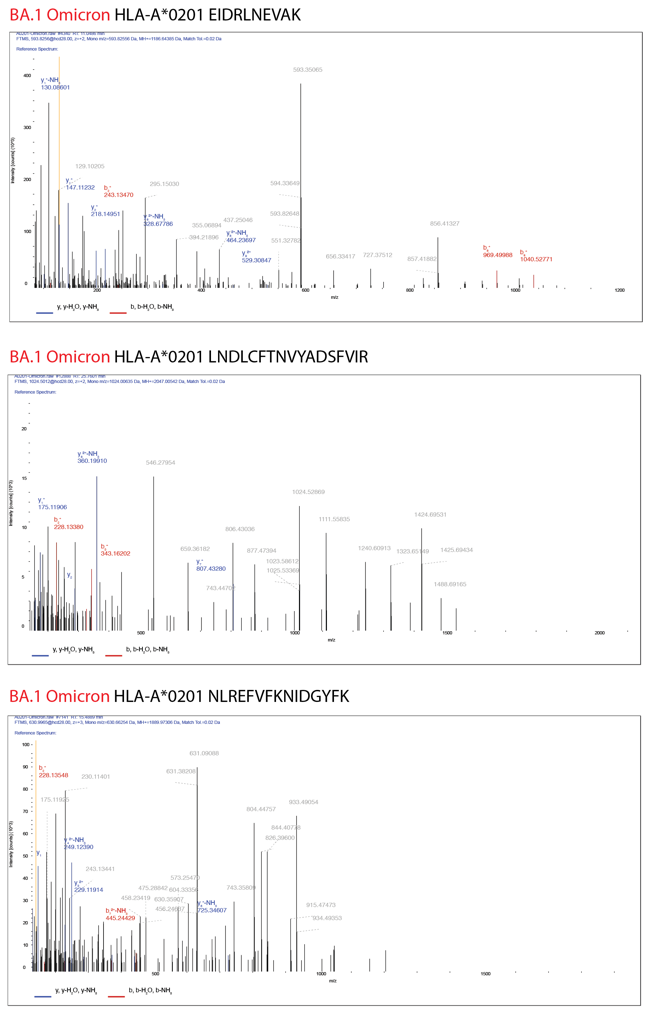


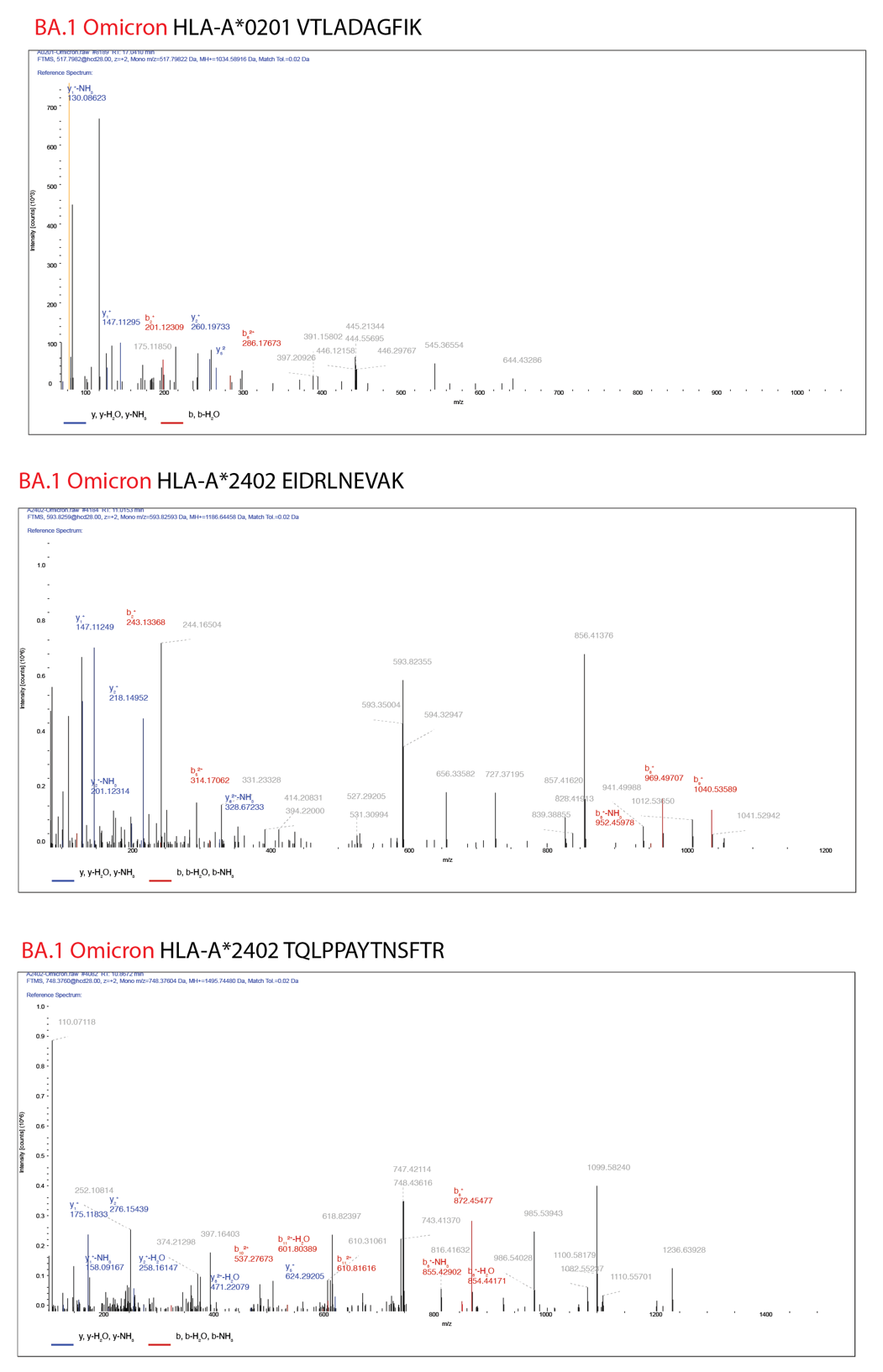


Figure. S5.

LC-MS/MS spectrum of the BA.1 Omicron spike protein peptides EIDRLNEVAK, LNDLCFTNVYADSFVIR, NLREFVFKNIDGYFK, VTLADAGFIK identified in Cos-7-HLA-A*02:01 cells; EIDRLNEVAK, TQLPPAYTNSFTR identified in Cos-7-HLA-A*24:02 cells. Spectrum images of these peptides were extracted from Proteome Discoverer 2.4.

Figure. S6.


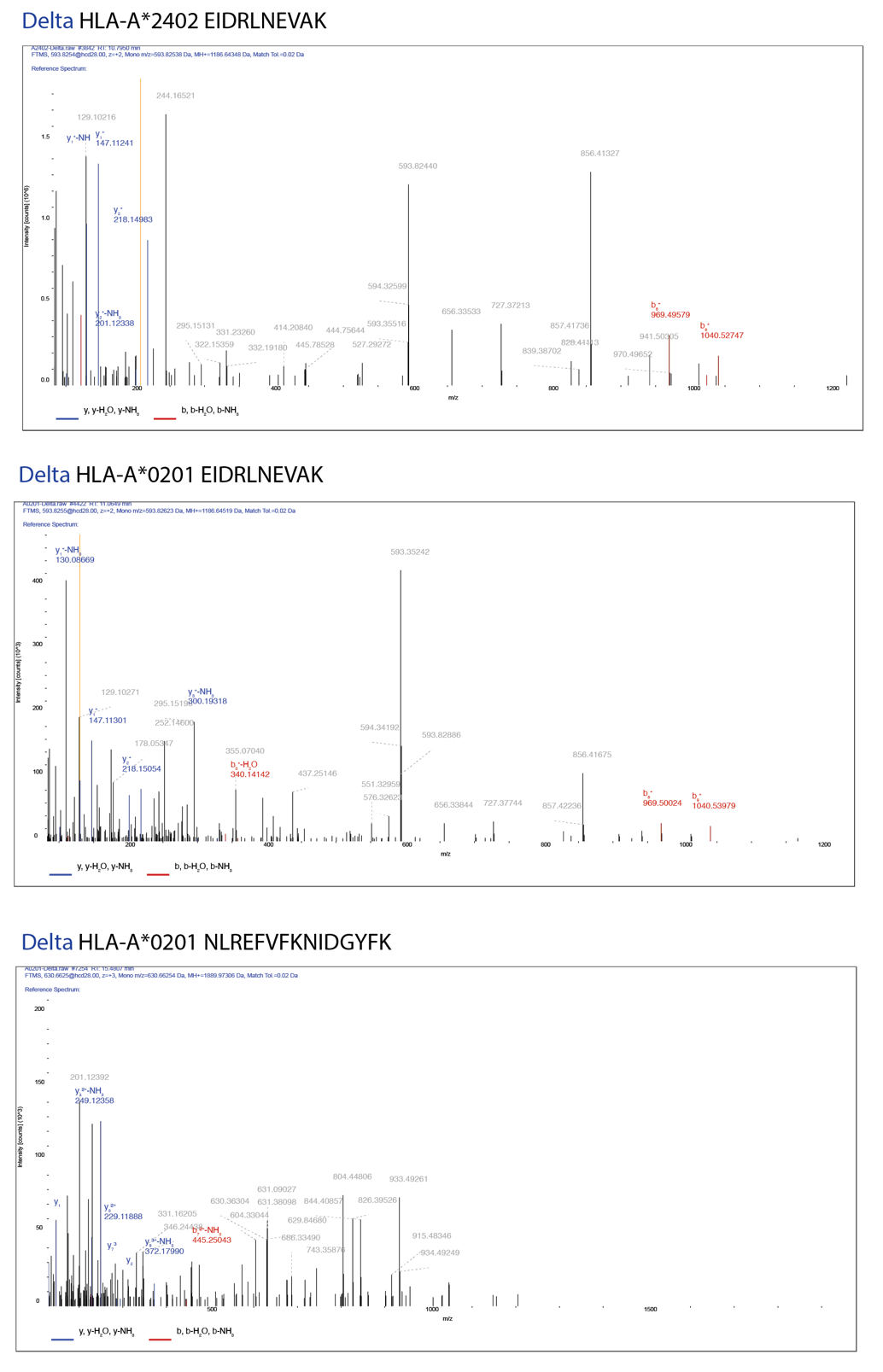


Figure. S6.

LC-MS/MS spectrum of the Delta spike protein peptides EIDRLNEVAK, NLREFVFKNIDGYFK identified in Cos-7-HLA-A*02:01 cells; and EIDRLNEVAK identified in Cos-7-HLA-A*24:02 cells. Spectrum images of these peptides were extracted from Proteome Discoverer 2.4.

Figure. S7.


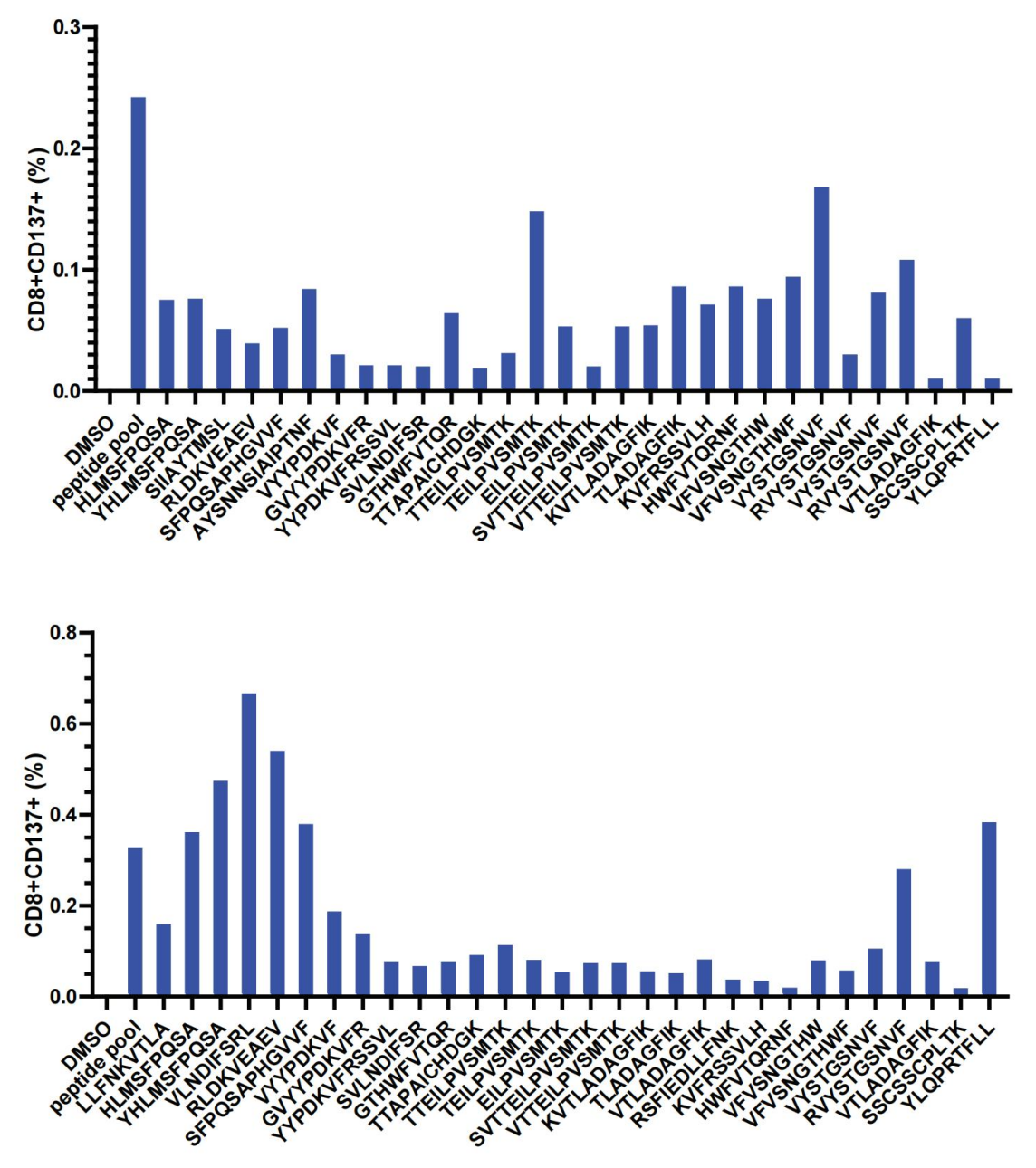


Figure. S7.

FACS results of T cell stimulation assays. Peptides from MS-based Immunopeptidome were used for T cell stimulation. The percentage of CD8+CD137+ were measured for antigen-specific T cells. Shown are results of two 3-dose vaccinees.

Figure. S8.


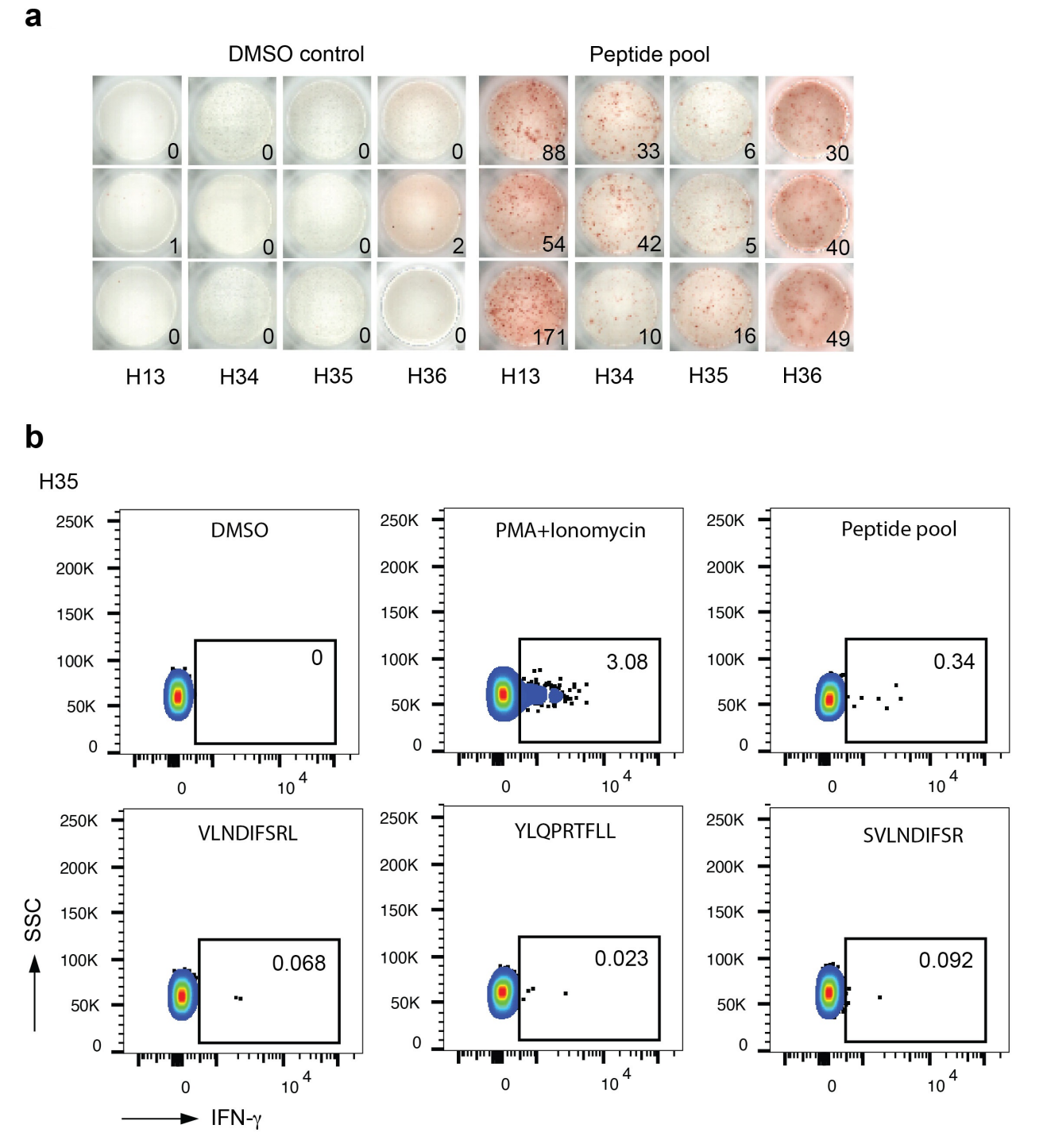


Figure. S8.

**a,b** The results of in vitro T cell stimulation with Elispot (**a**) or flow cytometry (**b**).

Figure. S9.


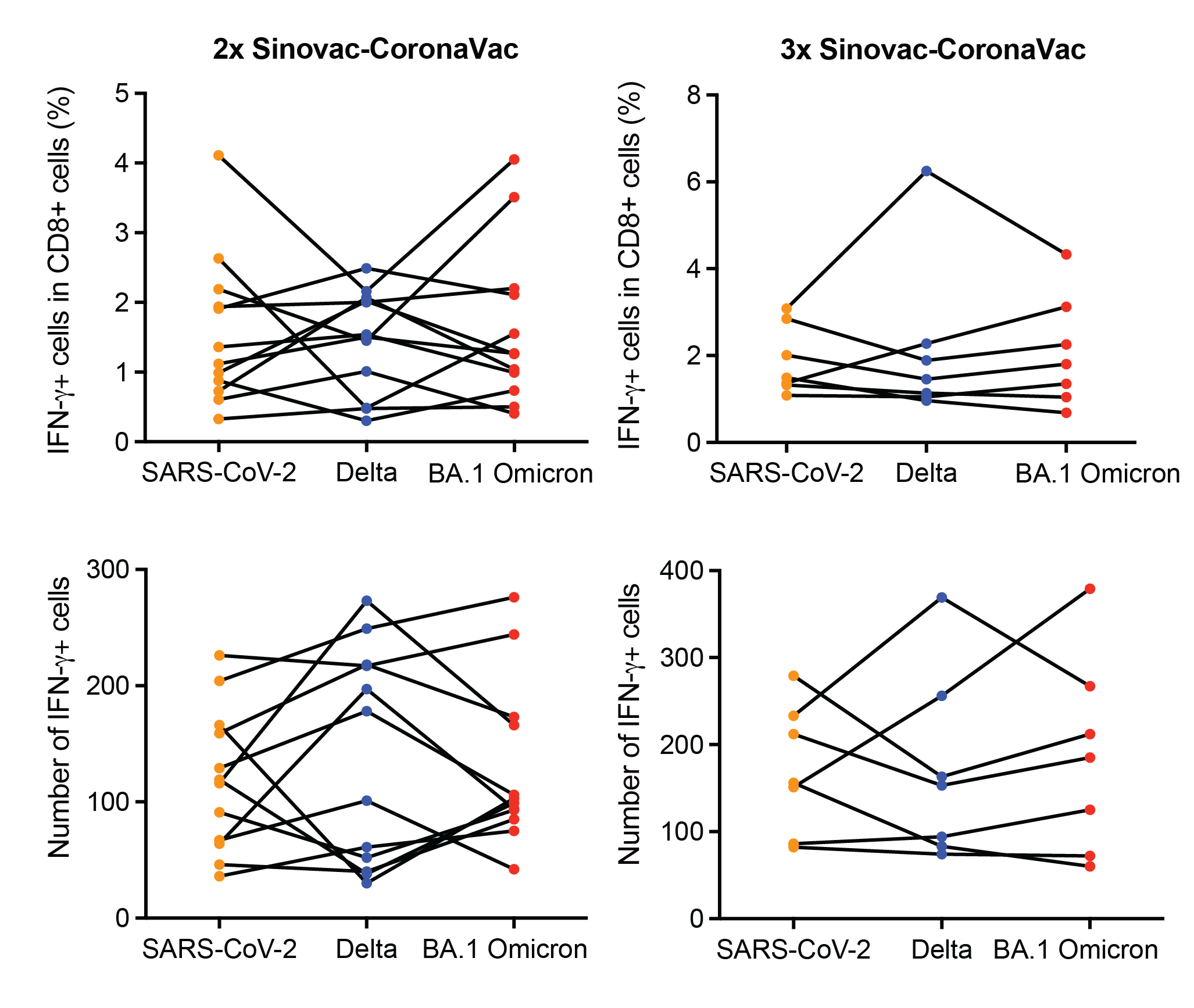


Figure. S9.

(Top) The percentage of IFN-$\gamma$ cells in total CD8+ T cells in 2-doses vaccinees (n=12) and 3-doses vaccinees (n=7). (Bottom) The absolute number of IFN-$\gamma$ cells in total CD8+ T cells in 2-doses vaccinees (n=12) and 3-doses vaccinees (n=7). p value was determined by the Wilcoxon rank sum test.

Figure. S10.


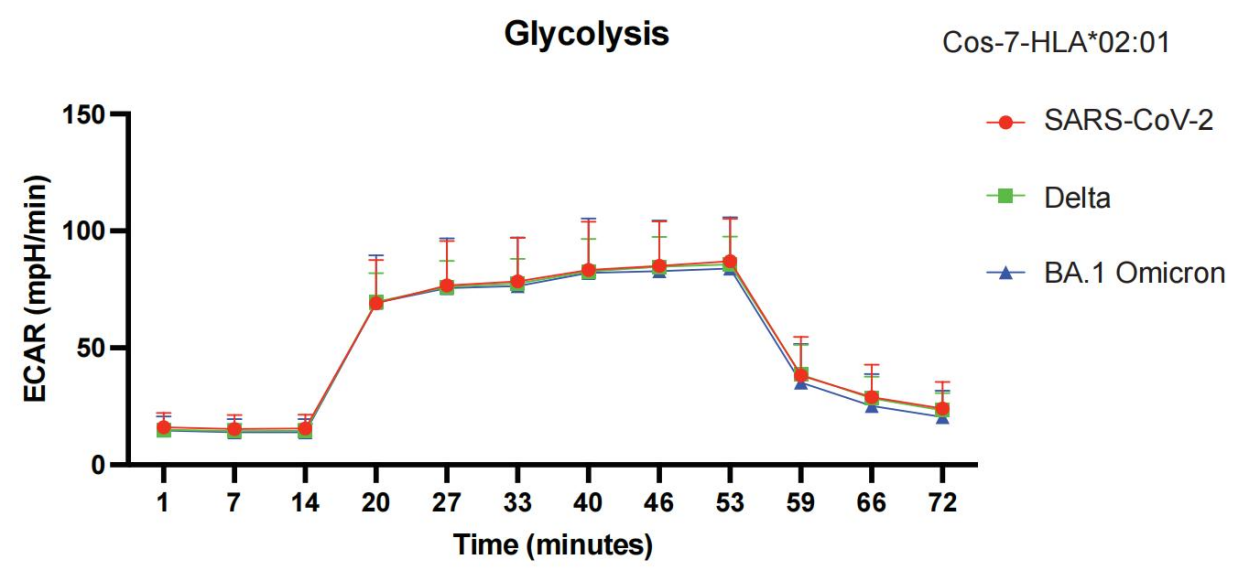


Figure. S10.

Real-time measurement of extracellular acidification rate (ECAR) in T cells co-cultured with SARS-CoV-2 spike gene lentivirus (SARS-CoV-2, Delta, Omicron) infected-Cos-7-HLA-A*02:01 cells (n=3).

Figure. S11.


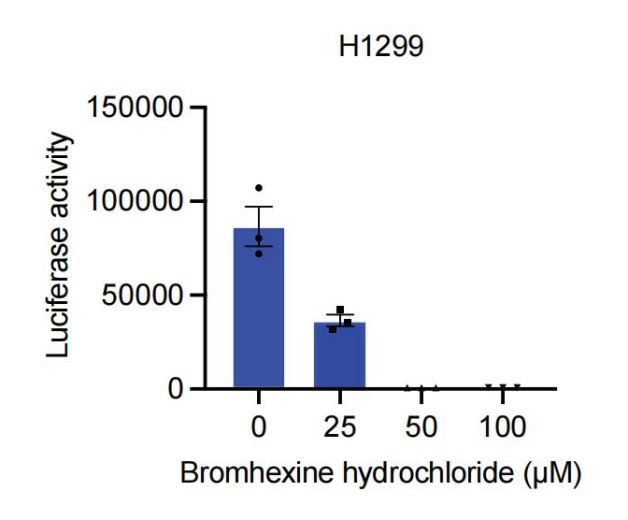


Figure. S11.

The ability of Bromhexine hydrochloride (a specific inhibitor of TMPRSS2) to inhibit SARS-CoV-2 pseudovirus infection for H1299 cells was evaluated by Dual-Luciferase Reporter System (n=3). Data are represented as mean ± SEM.

Figure. S12.


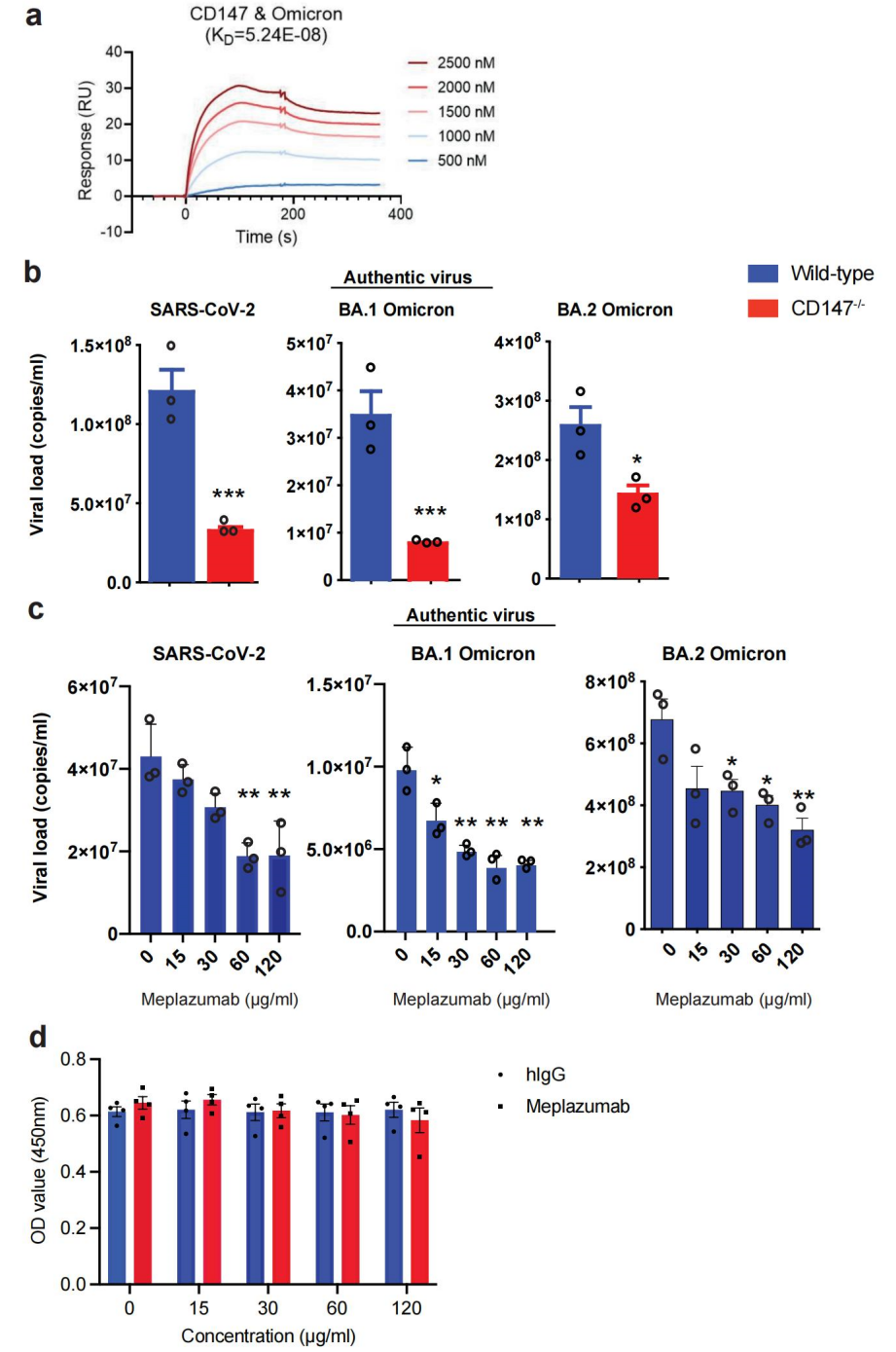


Figure. S12.

Inhibition of Omicron by meplazumab. **a** The interaction of CD147 and Omicron RBD detected by SPR assay. **b** SARS-CoV-2, BA.1 Omicron and BA.2 Omicron infected VeroE6 cells or CD147^-/-^ VeroE6 cells for 48 hours, and RNA was collected for viral RNA detection by RT-qPCR (n=3, two-tailed student t test). **c** RT-qPCR showed that meplazumab inhibits the infection of VeroE6 cells by authentic SARS-CoV-2, BA.1 Omicron and BA.2 Omicron. **d** The effect of meplazumab on VeroE6 cell proliferation was performed by CCK-8 assay, the concentrations of meplazumab were 15, 30, 60, and 120 μg/ml, and hIgG antibody was used as a control (n=3, Two-tailed student t test). Data are represented as mean ± SEM, *: p<0.05, **: p<0.01, ***: p<0.001.

Figure. S13.


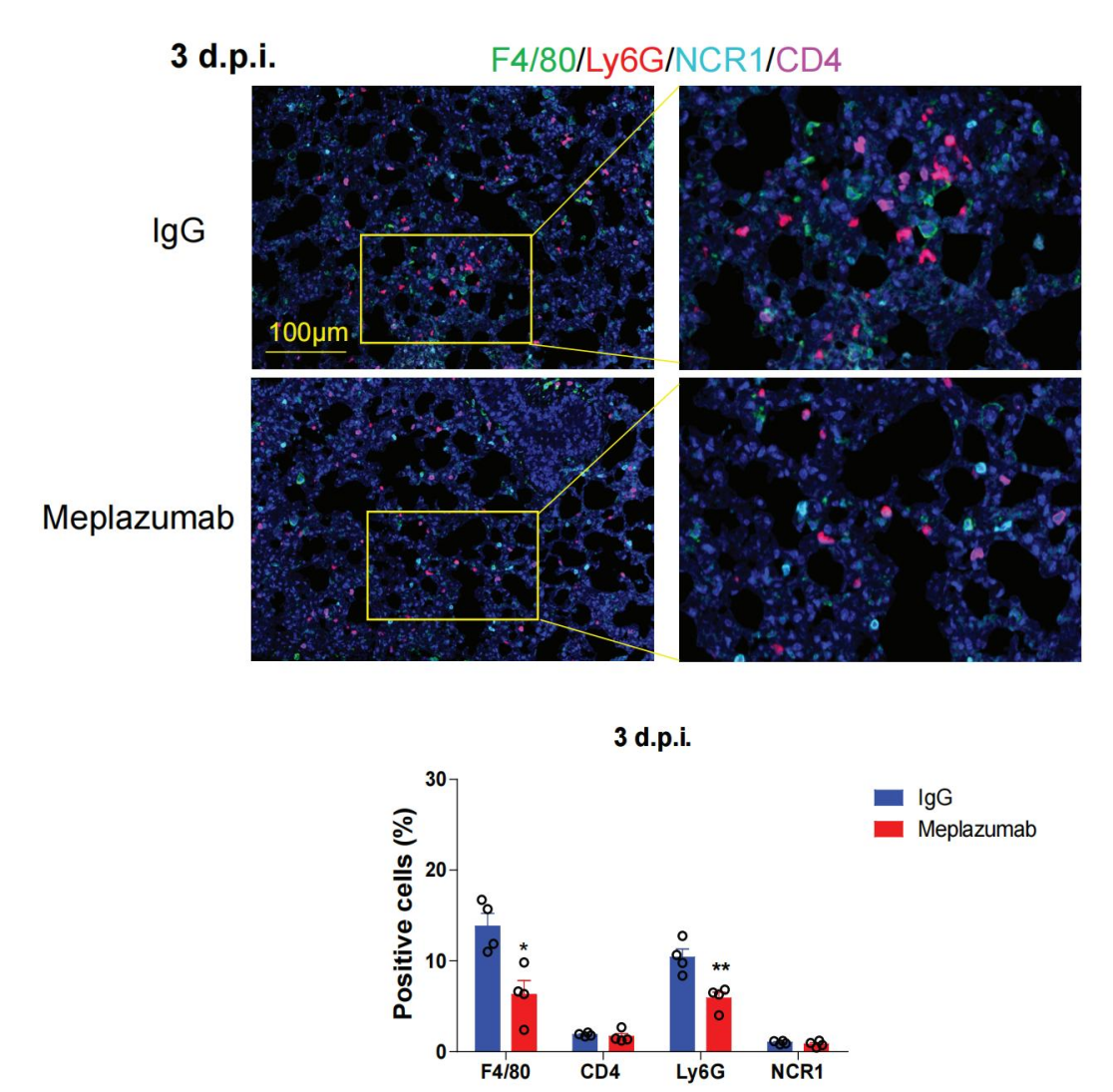


Figure. S13.

Multiplex immunofluorescence staining for macrophages, neutrophils, NK cells and CD4^+^ T cells in IgG and meplazumab group at 3 d.p.i. Data are represented as mean ± SEM, p values were determined by two-tailed student t test, *: p<0.05, **: p<0.01.

Figure. S14.


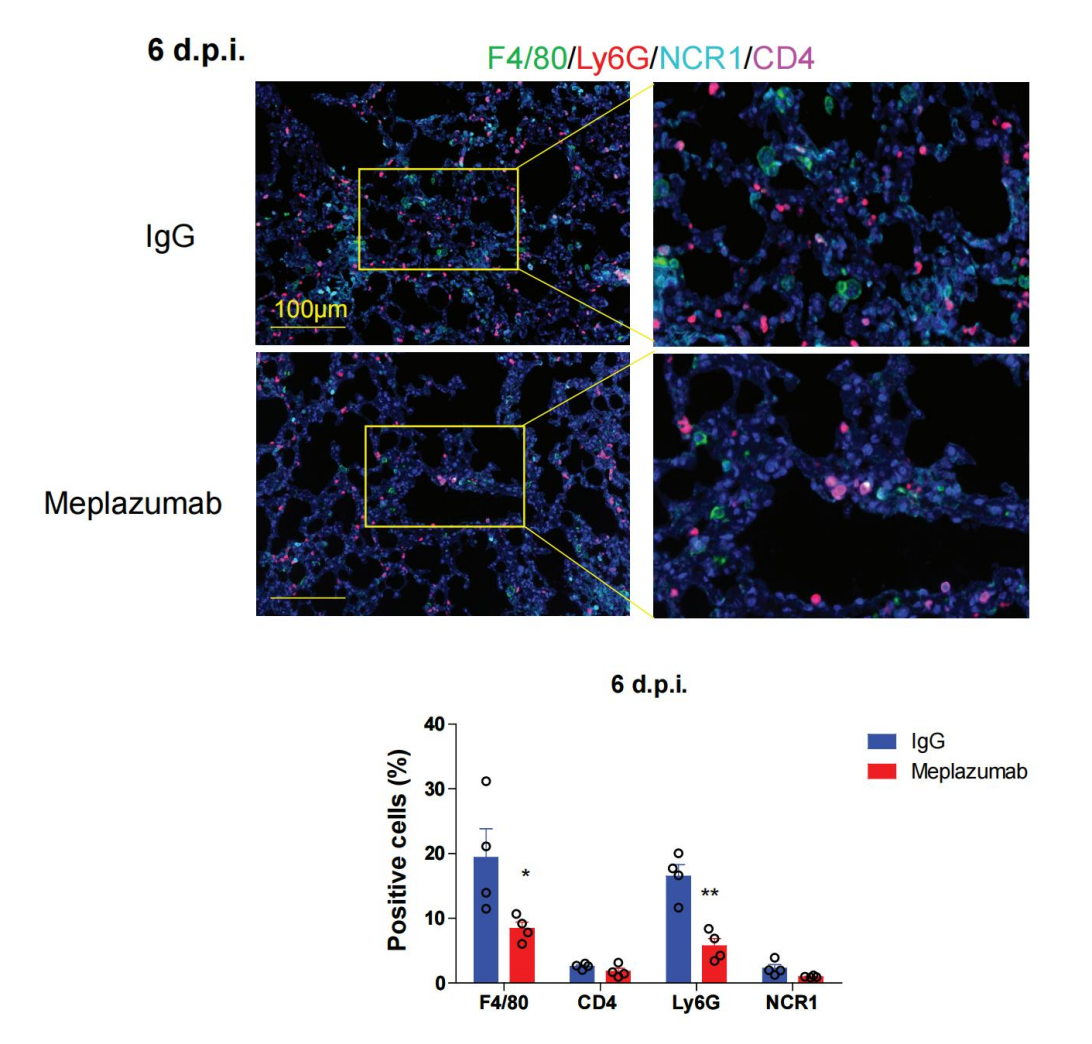


Figure. S14.

Multiplex immunofluorescence staining for macrophages, neutrophils, NK cells and CD4^+^ T cells in IgG and meplazumab group at 6 d.p.i. Data are represented as mean ± SEM, p values were determined by two-tailed student t test, *: p<0.05, **: p<0.01.

Figure. S15.


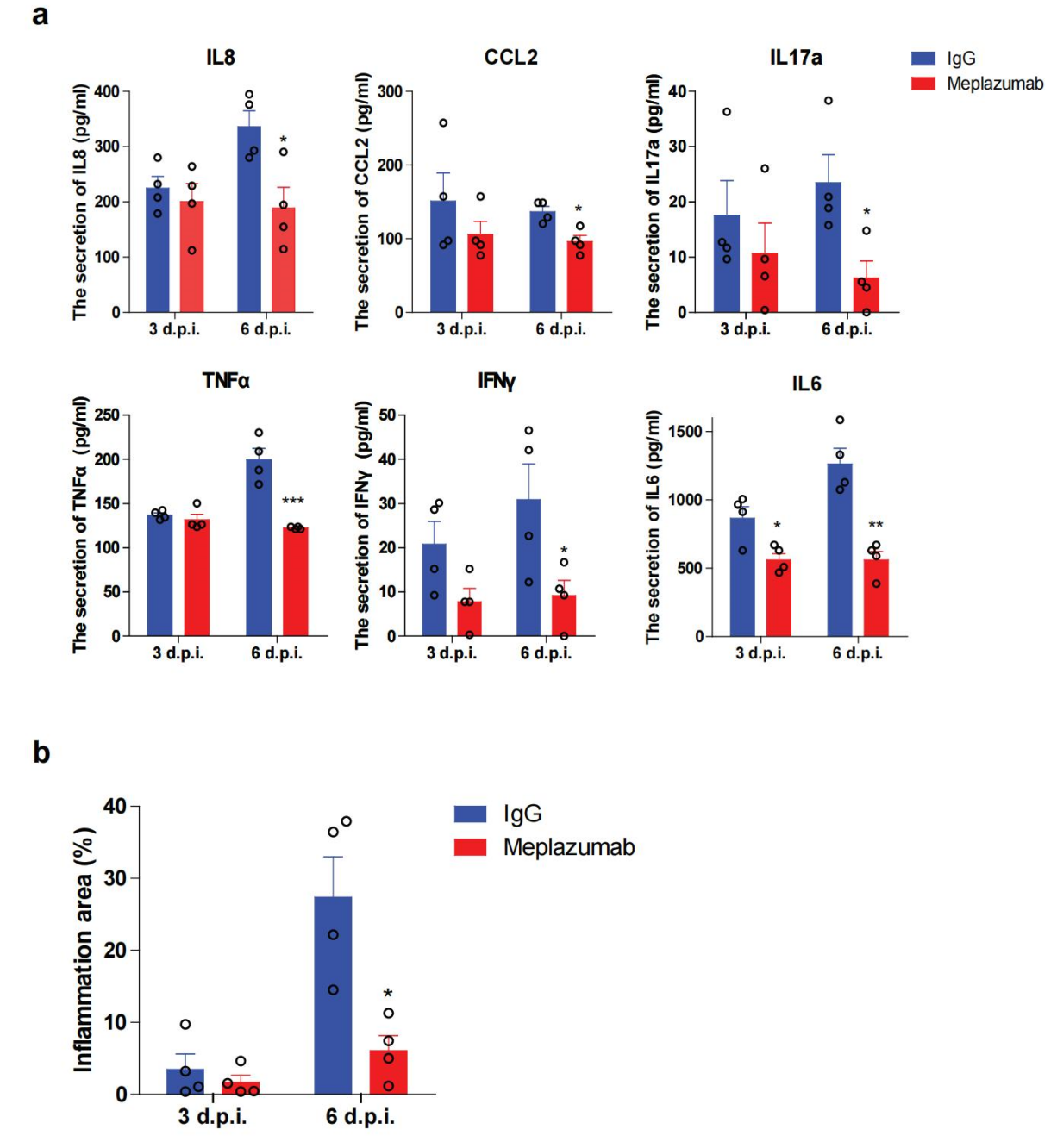


Figure. S15.

**a** Serum cytokines/chemokines (IL8, CCL2, IL17a, TNF-$\alpha$, IFN-$\gamma$ and IL-6) with ELISA detection in IgG and meplazumab group at 3 d.p.i. and 6 d.p.i. **b** The inflammatory area of H&E staining was quantified in lung tissues of IgG and meplazumab groups. The quantification of inflammatory conditions in H&E sections was calculated as follows: the inflammatory area/total area (%). Data are represented as mean ± SEM, p values were determined by two-tailed student t test, *: p<0.05, **: p<0.01, ***: p<0.001.

**Table S1. Information of COVID-19 convalescent patients.**

| Case | HLA-A | Exposure time (days) | Vaccinated | Blood collection time (days) |
| --- | --- | --- | --- | --- |
| P1 | 24:02/30:03 | 14 |  |  |
| P2 | 01:01/11:02 | 23 |  |  |
| P3 | 02:07/11:01 | 12 |  |  |
| P4 | 02:01/32:01 | 22 | Yes | 19. 17±5.78 |
| P5 | 11:01/11:02 | 17 |  |  |
| P6 | 11:01/30:01 | 17 |  |  |
| P7 | 11:01/30:01 | 14 |  |  |
| P8  P9 | 11:01/24:02 11:01/11:01 | 15  26 | No | 20.50±6.95 |
| P10 | 11:01/11:02 | 27 |  |  |

**Table S2. Information of vaccinees.**

| Case | HLA-A alleles | Vaccination  dose | Date of latest  vaccination | Blood collection  date |
| --- | --- | --- | --- | --- |
| H1 | Other HLA-A alleles | 2-dose | 2021/5/20 | 2022/3/15 |
| H2 | Other HLA-A alleles | 2-dose | 2021/6/20 | 2022/3/15 |
| H3 | Other HLA-A alleles | 2-dose | 2021/6/17 | 2022/3/15 |
| H4 | Other HLA-A alleles | 2-dose | 2021/6/25 | 2022/3/15 |
| H5 | 11:01 | 2-dose | 2021/6/17 | 2022/3/15 |
| H6 | 11:01 | 2-dose | 2021/6/19 | 2022/3/15 |
| H7 | 02:01/24:02 | 2-dose | 2021/6/13 | 2022/3/15 |
| H8 | 02:01/11:01 | 2-dose | 2021/5/15 | 2022/3/15 |
| H9 | 11:01 | 2-dose | 2021/4/16 | 2022/3/15 |
| H10 | 11:01 | 2-dose | 2021/6/27 | 2022/3/15 |
| H11 | 02:01 | 2-dose | 2021/6/14 | 2022/3/15 |
| H12 | Other HLA-A alleles | 2-dose | 2021/6/29 | 2022/3/15 |
| H13 | 02:01/24:02 | 3-dose | 2022/2/13 | 2022/3/15 |
| H14 | 24:02 | 3-dose | 2022/2/14 | 2022/3/15 |
| H15 | 02:01 | 3-dose | 2022/2/17 | 2022/3/15 |
| H16 | 02:01 | 3-dose | 2021/12/6 | 2022/3/15 |
| H17 | 11:01/24:02 | 3-dose | 2021/12/8 | 2022/3/15 |
| H18 | 11:01 | 3-dose | 2022/3/5 | 2022/3/15 |
| H19 | 11:01 | 3-dose | 2022/2/26 | 2022/3/15 |
| H20 | 11:01 | 3-dose | 2022/2/24 | 2022/3/15 |
| H21 | 24:02 | 3-dose | 2022/2/15 | 2022/3/15 |
| H22 | Other HLA-A alleles | 3-dose | 2022/2/19 | 2022/3/15 |
| H23 | 24:02 | 3-dose | 2021/10/19 | 2022/3/15 |
| H24 | 02:01/11:01 | 3-dose | 2021/11/30 | 2022/3/15 |
| H25 | 02:01/24:02 | 3-dose | 2022/2/13 | 2022/3/15 |
| H26 | 24:02 | 3-dose | 2022/2/14 | 2022/3/15 |
| H27 | 02:01 | 3-dose | 2022/2/17 | 2022/3/15 |
| H28 | 02:01 | 3-dose | 2021/12/6 | 2022/3/15 |
| H29 | 02:01 | 3-dose | 2021/12/3 | 2022/3/15 |
| H30 | 11:01/24:02 | 3-dose | 2021/12/8 | 2022/3/15 |
| H31 | 11:01 | 3-dose | 2022/3/5 | 2022/3/15 |
| H32 | 11:01 | 3-dose | 2022/2/26 | 2022/3/15 |
| H33 | 11:01 | 3-dose | 2022/2/24 | 2022/3/15 |
| H34 | 24:02/02:01 | 3-dose | 2022/2/15 | 2022/3/15 |
| H35 | 24:02/02:01 | 3-dose | 2021/10/19 | 2022/3/15 |
| H36 | 02:01 | 3-dose | 2021/10/19 | 2022/3/15 |
